# Identification and Heterologous Expression of the Chaxamycin Biosynthesis Gene Cluster from Streptomyces leeuwenhoekii

**DOI:** 10.1128/AEM.01039-15

**Published:** 2015-08-07

**Authors:** Jean Franco Castro, Valeria Razmilic, Juan Pablo Gomez-Escribano, Barbara Andrews, Juan A. Asenjo, Mervyn J. Bibb

**Affiliations:** a Centre for Biotechnology and Bioengineering, Department of Chemical Engineering and Biotechnology, Universidad de Chile, Santiago, Chile; b Department of Molecular Microbiology, John Innes Centre, Norwich Research Park, Norwich, United Kingdom

## Abstract

Streptomyces leeuwenhoekii, isolated from the hyperarid Atacama Desert, produces the new ansamycin-like compounds chaxamycins A to D, which possess potent antibacterial activity and moderate antiproliferative activity. We report the development of genetic tools to manipulate S. leeuwenhoekii and the identification and partial characterization of the 80.2-kb chaxamycin biosynthesis gene cluster, which was achieved by both mutational analysis in the natural producer and heterologous expression in Streptomyces coelicolor A3(2) strain M1152. Restoration of chaxamycin production in a nonproducing Δ*cxmK* mutant (*cxmK* encodes 3-amino-5-hydroxybenzoic acid [AHBA] synthase) was achieved by supplementing the growth medium with AHBA, suggesting that mutasynthesis may be a viable approach for the generation of novel chaxamycin derivatives.

## INTRODUCTION

Antibiotic resistance is rapidly becoming a worldwide medical problem that requires urgent attention (1–3). Historically, natural products produced by actinomycetes and fungi have been the major source of clinically used antibiotics (4). However, the repeated rediscovery of known chemical entities from natural sources has led to a marked decline in the discovery of novel microbial antibiotics with clinical utility (5, 6). One potential solution to this problem is to screen microorganisms isolated from previously little-scrutinized ecological niches, and bioprospecting of unexplored environments has indeed already resulted in the discovery of novel microorganisms and new chemical scaffolds (4, 6).

**FIG 1:**
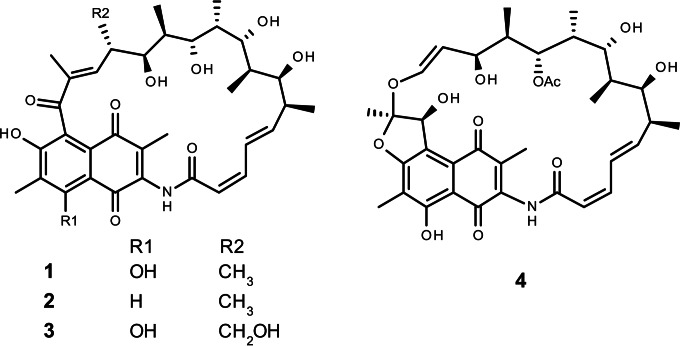
Structures of chaxamycins A to D (compounds 1 to 4).

In 2004, a considerable number of new actinomycetes were, surprisingly, isolated from the hyperarid Atacama Desert, the oldest and driest desert on Earth, located in northern Chile (7, 8). Strikingly, some of these strains produce novel chemical structures with potent biological activity (9–11). One of the strains isolated from this environment is Streptomyces leeuwenhoekii DSM 42122 (previously known as Streptomyces strain C34^T^), isolated from the Chaxa Lagoon (7, 12). It produces the new polyketide antibiotics called the chaxamycins (9) and the chaxalactins (10). Chaxamycin A to D (compounds 1 to 4 in Fig. 1) are four new naphthalenic ansamycin-type polyketides derived from a 3-amino-5-hydroxybenzoic acid (AHBA) starter unit. Chaxamycin D (compound 4) displays, through an unknown mechanism, highly selective antimicrobial activity against Staphylococcus aureus ATCC 25923 and against a panel of methicillin-resistant S. aureus (MRSA) strains (9), while chaxamycin A (compound 1) inhibits the intrinsic ATPase activity of the human Hsp90 protein, which is involved in cancer proliferation (9). The chaxamycins differ from other naphthalenic ansamycins, such as the rifamycins, naphthomycin, geldanamycin, and the herbimycins, in lacking a methyl group at the olefinic C-16 next to the amide bond present in all of these molecules.

The biosynthesis of ansamycin-type polyketides commences with priming of the polyketide synthase (PKS) with the precursor molecule AHBA. In the case of the rifamycin and other ansamycin-type polyketide gene clusters, a sub-gene cluster, located near the PKS, is largely responsible for the synthesis of AHBA (13). One of the enzymes encoded in the core of this AHBA gene cluster is AHBA synthase, which converts 5-deoxy-5-amino-3-dehydroshikimic acid (amino-DHS) into AHBA, the final step in its biosynthesis (14), and an AHBA synthase gene indeed was identified previously by PCR analysis of genomic DNA of S. leeuwenhoekii (9). In addition to AHBA, we predict that chaxamycin is synthesized from 10 additional extender units (three malonyl coenzyme As [malonyl-CoAs] and seven methylmalonyl-CoAs) by a modular type I PKS.

Recent improvements in next-generation sequencing technologies and genome mining have markedly accelerated the identification and characterization of gene clusters for specialized metabolites, many potentially encoding novel compounds with antimicrobial activity. In this work, we describe the application of these technologies to identify and partially characterize the chaxamycin biosynthesis gene cluster of S. leeuwenhoekii.

## MATERIALS AND METHODS

### Strains, culture conditions, and general methods.

The bacterial strains used and generated during this study are listed in Table 1. Escherichia coli and Streptomyces strains were cultured, maintained, and manipulated genetically following the methods of Sambrook et al. (15) and Kieser et al. (16), respectively. The plasmids and oligonucleotides used or constructed during this work are listed in Table 2 and Table 3, respectively. Mobilization of phage P1-derived artificial chromosome (PAC) clones into Streptomyces strains was performed as described previously (17). Molecular biology enzymes, reagents, and kits were used according to the manufacturer's instructions. High-fidelity PCR amplification was performed with Phusion or Q5 DNA polymerase following the manufacturer's instructions (NEB, Ipswich, MA) with a nucleotide proportion of 15A:15T:35G:35C to improve the amplification efficiency of high-moles-percent G+C Streptomyces DNA. 3-Amino-5-hydroxybenzoic acid (AHBA) was purchased from Sigma (catalog no. PH011754) and dissolved in dimethyl sulfoxide.

**TABLE 1:** Bacterial strains used in this study

Species and strain	Description	Reference or source
Escherichia coli		
DH5α	Strain used for routine cloning	65
DH10B	Strain used for routine cloning	65
ET12567/pUZ8002	Methylation-deficient strain used for conjugation with *Streptomyces*; pUZ8002 provides conjugation machinery	66 (pUZ8002, J. Wilson and D. Figurski, unpublished)
TOP10/pR9406	Strain used for routine cloning carrying conjugation plasmid pR9406	pR9406, A. Siddique and D. Figurski, unpublished.
Streptomyces coelicolor		
M1152	M145 Δ*act* Δ*red* Δ*cpk* Δ*cda rpoB*(C1298T)	67
M1650	M1152 carrying pIJ12853	This work
Streptomyces leeuwenhoekii		
Wild-type strain		12
M1653	S. leeuwenhoekii Δ*cxmK*::*neo*	This work
M1655	S. leeuwenhoekii/pGUS	This work
M1656	S. leeuwenhoekii/pIJ10740	This work

**TABLE 2:** Plasmids used and constructed during this study

Plasmid	Description	Reference or source
pBluescript II KS(+)	General cloning vector	68
pKC1132	Cloning vector, conjugative (*oriT* from RK2)	69
pSET152	Cloning vector, conjugative (*oriT* from RK2), integrative (phiC31 *attP*)	69
pTC192-Km	Source of *neo* (kanamycin resistance gene)	70
pGM1190	pSG5 derivative, *tsr*, *aac*(*3*)*IV*, *oriT*, *to* terminator, P_tipA_, RBS, *fd* terminator	40 (pGM1190, G. Muth, unpublished)
pESAC13	PAC vector (P1 phage replicon) for genomic library construction; conjugative (*oriT* from RK2), integrative (phiC31 *attP*), *tsr*, *neo*, P1 *rep*, *sacB*	27 (pESAC13, M. Sosio, unpublished)
pGUS	*gusA* (β-glucuronidase gene, codon-optimized for streptomycetes) in pSET152	38
pIJ10740	pGUS derivative with ermE*p driving *gusA* transcription	Morgan Feeney, unpublished
pIJ10257	Expression vector for *Streptomyces*, with ermE*p, *hyg*, conjugative (*oriT* from RK2), integrative (phiC31 *attP*)	71
pIJ12850	Derivative of pGM1190 with JFC010/JFC009-*neo*-JFC011/JFC012 fragments; for deletion of *cxmK*	This work
pIJ12851	Derivative of pKC1132 with JFC010/JFC009-*neo*-JFC011/JFC012 fragments; for deletion of *cxmK*	This work
pIJ12857	Derivative of pKC1132 with JFC010/JFC009 fragment; for deletion of *cxmK*	This work
pIJ12858	Derivative of pKC1132 with JFC011/JFC012 fragment; for deletion of *cxmK*	This work
pIJ12859	Derivative of pKC1132 with JFC010/JFC009-JFC011/JFC012 fragment; for deletion of *cxmK*	This work
pIJ12860	Derivative of pGM1190 with JFC010/JFC009-JFC011/JFC012 fragment; for deletion of *cxmK*	This work
pIJ12861	pBluescript II KS(+) derivative with JFC026/JFC034 fragment; for cloning of *cxmK*	This work
pIJ12852	pIJ10257 derivative with *cxmK*; for complementation of deletion mutant	This work
pIJ12853	Derivative of pESAC13; contains 145 kb of S. leeuwenhoekii chromosome, including the chaxamycin biosynthesis gene cluster	This work

**TABLE 3:**
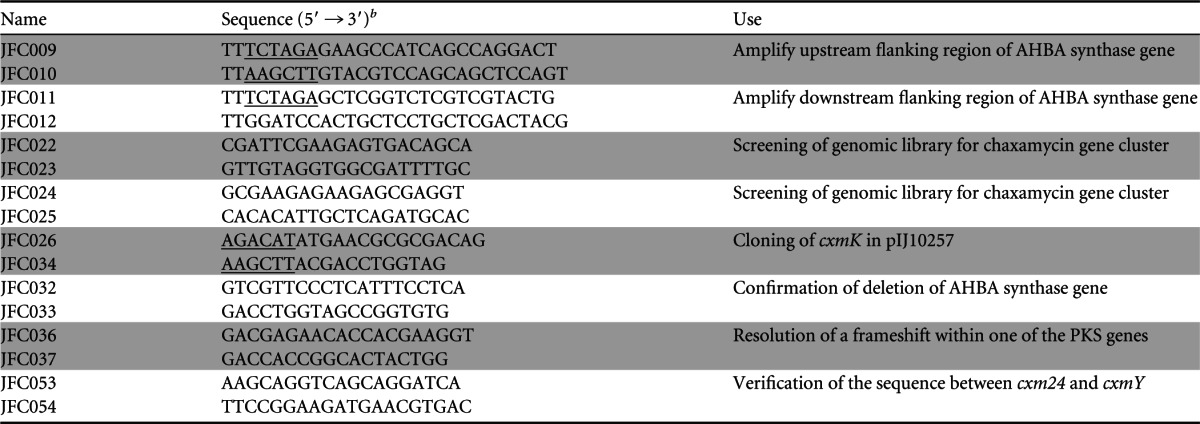
Oligonucleotides used in this study^*a*^

a Oligonucleotide pairs are shown in consecutive shaded or unshaded rows, and the information in the “Use” column applies to both relevant oligonucleotides.

b Incorporated restriction sites are underlined.

### Specific methods for S. leeuwenhoekii.

Spore stocks of S. leeuwenhoekii were prepared following standard methods (16) from cultures grown on soy flour-mannitol (SFM) agar medium at 37°C for 5 to 7 days. Mobilization of plasmids into S. leeuwenhoekii was performed as established for Streptomyces coelicolor A3(2) by conjugation from E. coli ET12567 (carrying either pUZ8002 or pR9406) using 10^8^ spores that were heat shocked in 2× YT medium at 50°C for 10 min; the treated spores were allowed to cool to room temperature before mixing them with the E. coli cells, and the conjugation mixtures were plated on SFM agar medium supplemented with 10 mM MgCl_2_ and 10 mM CaCl_2_.

### Production and measurement of chaxamycins.

S. leeuwenhoekii strains were grown in modified ISP2 medium (containing, per liter, 4.0 g of yeast extract, 10.0 g of malt extract, and 10.0 g of glycerol [pH 7.20] [9]). For heterologous production of chaxamycin, S. coelicolor strains were cultured in R3 medium (18) [containing, per liter, 10 g of glucose, 5 g of yeast extract, 100 mg of Casamino Acids, 3 g of proline, 10 g of MgCl_2_ · 6H_2_O, 4 g of CaCl_2_ · 2H_2_O, 200 mg of K_2_SO_4_, 50 mg of KH_2_PO_4_, 5.6 g of *N*-tris(hydroxymethyl)methyl-2-aminoethanesulfonic acid (TES), and trace elements (as described for R2 medium in reference 16) and adjusted to pH 7.2 with NaOH]. All strains were cultured in 250-ml Erlenmeyer flasks that had been treated with 5% dimethyldichlorosilane (DMDCS) in toluene (Sylon CT; Supelco, catalog number 33065-U) to deter the mycelium from sticking to the glass; a stainless steel spring was placed at the bottom of each flask to improve aeration. Seed cultures were inoculated with 10^8^ spores and incubated at 30°C with orbital shaking (250 rpm) for 24 to 48 h. Mycelium from the seed cultures was harvested by centrifugation, washed with 0.9% NaCl, resuspended in fresh medium, and used to inoculate production cultures to a final optical density at 600 nm (OD_600_) of 0.2; production cultures were incubated at 30°C with orbital shaking (250 rpm). Samples were harvested at the indicated times: 1 or 2 ml of homogenized culture was pipetted into a clean Eppendorf tube and centrifuged at 13,000 rpm at 4°C for 10 min, the supernatant was moved to a new Eppendorf tube and centrifuged again at 13,000 rpm at 4°C for 10 min before carefully pipetting 0.5 ml of the supernatant into a high-pressure liquid chromatography (HPLC) vial, the mycelial pellet was extracted with 0.5 ml of methanol and centrifuged at 13,000 rpm at 4°C for 10 min, and 0.3 ml of the methanol extract was pipetted into an HPLC vial containing 0.3 ml of water. Chaxamycins were detected in both the supernatants and the mycelial extracts, but larger amounts were always found in the supernatants, and thus only these fractions were analyzed in most experiments. When analyzing total chaxamycin production in a single HPLC run, both fractions were mixed in a 1:1 ratio (thus injecting samples in 50% methanol). Samples were analyzed on a Shimadzu liquid chromatography-mass spectrometry (LC-MS) system equipped with a NexeraX2 liquid chromatograph (LC30AD) fitted with a Prominence photodiode array detector (SPD-M20A) and an LCMS-IT-ToF mass spectrometer; samples (typically 5 μl) were injected in a Kinetex XB C_18_ 2.6-μm, 100-Å, 50- by 2.10-mm column (part no. 00B-4496-AN; Phenomenex, USA) fitted with a KrudKatcher Ultra HPLC in-line filter (part no. AF0-8497; Phenomenex, USA) and eluted at a flow rate of 0.6 ml per min with a gradient of 0.1% formic acid in water (mobile phase A) and methanol (mobile phase B) as follows (minute, percentage of B reached at that minute): 0 min, 2% B; 1 min, 2% B; 8 min, 100% B; 9.3 min, 100% B; 9.5 min, 2% B; and 11.2 min, 2% B for equilibration. The column was kept at 40°C. Mass spectrometry detection was performed in negative ion mode since all of the chaxamycin species were detected with higher sensitivity than in positive mode. Purified chaxamycin standards for compounds 1, 2, and 3 were kindly provided by Mostafa Rateb and Marcel Jaspars (University of Aberdeen, Scotland). Data acquisition and analysis were performed with LCMSsolutions version 3 (Shimadzu); spectrum visualization was also performed with Mass++ version 2.7.2 (http://www.first-ms3d.jp/english/). The (M − H)^−^ ions detected for the chaxamycin species were as follows: compound 1, *m/z* 638.29; compound 2, *m/z* 622.29; compound 3, *m/z* 654.29; and compound 4, *m/z* 682.29.

### DNA sequencing and bioinformatic analysis.

The sequencing and assembly of the genome sequence of S. leeuwenhoekii has been reported elsewhere (19). Confirmatory Sanger sequencing was performed using the Eurofins Genomics service (Ebersberg, Germany) and analyzed using the Staden package (20) version 2.0.0b9 (http://staden.sourceforge.net). BLAST (21) searches were performed at the NCBI server. Local BLAST searching was performed using NCBI package 2.2.26+ (22) and SequenceServer (23) or prfectBLAST (24). Automatic primer design was carried out using the Primer3Plus web server (25). DoBISCUIT (http://www.bio.nite.go.jp/pks/top) (Database of Biosynthesis clusters Curated and Integrated) (26) was used to obtain curated and annotated versions of ansamycin-related gene clusters. The sequence of phage P1-derived artificial chromosome (PAC) clone 2-11L (named pIJ12853), selected from a library of S. leeuwenhoekii genomic DNA made in pESAC13 (E. coli-Streptomyces artificial chromosome [27]; http://www.biost.com/page/PAC.aspx) and containing the chaxamycin biosynthesis gene cluster (see “PAC library construction and PCR screening for the chaxamycin gene cluster” below) was obtained using a Roche 454-Junior sequencer (commissioned from the DNA sequencing facility in the Department of Biochemistry, University of Cambridge, Cambridge, United Kingdom). A high-quality sequence of the complete PAC insert was obtained by mapping the assembled contigs from the 454-Junior sequencing onto the genome sequence (19) using the Burrows Wheeler Aligner BWA-MEM algorithm with default parameters (28); the resulting BAM alignment file was processed with SAMTOOLS (29) and loaded into GAP5 (30) to manually produce a consensus sequence. This consensus was processed with Prodigal (31) to identify protein-coding sequences, automatically annotated using the BASys (32) web server (https://www.basys.ca/), and analyzed and curated with Artemis (33). Analysis of the sequence of the insert of pIJ12853 revealed that it corresponded to nucleotide (nt) positions 1151661 to 1305894 of the published S. leeuwenhoekii chromosome sequence (European Nucleotide Archive study accession number PRJEB8583) and contained many of the genes predicted to be required for chaxamycin biosynthesis (located between nt 1211049 and 1289829). GC Frame Plot analysis (34) performed within Artemis revealed a clear frameshift in the 454 sequence within one of the PKS genes. The corresponding region was PCR amplified using primers JFC036 and JFC037 and sequenced with the same primers by Sanger sequencing. Analysis revealed the presence of two close frameshifts (two missing Gs), and the consensus sequence was corrected accordingly (matching that obtained with Illumina MiSeq sequencing of the S. leeuwenhoekii genome [19]). GC Frame Plot analysis revealed possible frameshifts between approximate nucleotide positions 1214200 and 1215100. Indeed, BLASTX analyses (21) confirmed that this region had the potential to encode a type II thioesterase (see Discussion) but contained three frameshifts in a putative ancestral coding sequence that begins at nt 1215047 and ends at nt 1214242. To assess the quality of this sequence, the region was PCR amplified using primers JFC053 and JFC054 with pIJ12853 (see below) DNA as the template, and the fidelity of the sequence and the presence of the frameshifts were confirmed by Sanger sequencing using the same primers.

### PAC library construction and PCR screening for the chaxamycin gene cluster.

A genomic library of S. leeuwenhoekii DNA was constructed in pESAC13. Briefly, high-molecular-weight DNA was digested partially with BamHI and ligated with pESAC13 digested with the same enzyme, resulting in loss of pUC19 present in the original vector. The genomic library was screened for clones containing the chaxamycin biosynthesis gene cluster by PCR using two primer pairs, JFC022/JFC023 and JFC024/JFC025, designed to amplify the predicted ends of the gene cluster. One PAC clone (2-11L) was identified, named pIJ12853, and sequenced (see “DNA sequencing and bioinformatic analysis” above). Both library construction and screening were carried out by Bio S&T Inc. (Montreal, Canada).

### Construction of S. leeuwenhoekii M1653 (Δ*cxmK*::*neo*).

The AHBA synthase gene (*cxmK*) was deleted and replaced with the kanamycin resistance gene *neo* by double-crossover homologous recombination. Two DNA segments flanking *cxmK*, each about 1.7 kb in length, were PCR amplified with primer pairs JFC010/JFC009 (containing 5′ HindIII and XbaI sites, respectively) and JFC011/JFC012 (containing 5′ XbaI and BamHI sites, respectively) and cloned separately in pKC1132 that had been cleaved with HindIII plus XbaI or XbaI plus BamHI to generate pIJ12857 and pIJ12858, respectively. Each of the amplified fragments was verified by DNA sequencing. pIJ12858 was cleaved with XbaI and BamHI, and the 1.7-kb band was purified from an agarose gel and cloned into pIJ12857 cut with XbaI and BamHI to generate pIJ12859. The HindIII-BamHI insert of pIJ12859 was cloned in the self-replicative temperature-sensitive plasmid pGM1190 that had been cleaved with HindIII and BamHI to yield pIJ12860. Finally, *neo* was excised with XbaI from pTC192-km and cloned in the XbaI site of pIJ12860 to yield pIJ12850. This construct was transferred into S. leeuwenhoekii by conjugation, and kanamycin-resistant exconjugants were selected at 30°C, restreaked on SFM agar containing kanamycin, and cultivated at 37°C, a temperature at which pGM1190 cannot replicate, therefore forcing chromosomal integration of *neo* by either single or double homologous recombination. The resulting spores were plated on SFM agar without antibiotic selection and incubated at 30°C, and the resulting colonies were replicated consecutively onto SFM agar plates containing just kanamycin, just apramycin, or neither antibiotic, which were then incubated at 37°C. Colonies resistant to kanamycin but sensitive to apramycin were subjected to PCR analysis using primers (JFC032/JFC033), which flanked *cxmK*, and the required Δ*cxmK*::*neo* deletion mutant (M1653) was identified (see Fig. S7 in the supplemental material). Note that we resorted to using a derivative of the replicative, multicopy pGM1190 for gene replacement after attempts to use pIJ12851 (Table 2), a derivative of the nonreplicative pKC1132 containing the same deletion cassette as pIJ12850, failed to yield single-crossover recombinants, which possibly was a consequence of a relatively low recombination frequency in S. leeuwenhoekii. We hoped to compensate for any such deficiency by using the multicopy, replicative pGM1190 derivative.

### Construction of pIJ12852.

*cxmK* was amplified from S. leeuwenhoekii genomic DNA by high-fidelity PCR using primers JFC026 and JFC034 (containing 5′ NdeI and HindIII sites, respectively); the PCR product was blunt-end ligated into the SmaI site of pBluescript II KS(+) to yield pIJ12861, and the sequence of the PCR product was verified by sequencing with universal primers corresponding to the vector. *cxmK* was excised from pIJ12861 with NdeI and HindIII and inserted into pIJ10257 that had been cleaved with the same enzymes to generate pIJ12852 with *cxmK* transcribed from the constitutive ermE* promoter.

### Generation of illustrations.

Illustrations of LC-MS/MS data were generated using Microsoft Office Suite. ImageJ version 1.47 (http://imagej.nih.gov/ij) and Inkscape version 0.91 (https://inkscape.org) were used to create and edit drawings and illustrations.

## RESULTS

### Establishment of microbiological and genetic manipulation procedures for S. leeuwenhoekii.

S. leeuwenhoekii grew well on SFM, R2, and DNA agar media, with the most copious and rapid sporulation observed after growth for 4 to 6 days at 30°C on SFM agar (see Fig. S1 in the supplemental material). Since S. leeuwenhoekii was isolated from the Atacama Desert (Chile), we compared growth at 30°C, 37°C, and 43°C. Growth and sporulation occurred more rapidly with increasing temperature, with sporulation occurring at 37°C and 43°C after just 2 days of cultivation (see Fig. S2 in the supplemental material). Consequently, we used 37°C to prepare spore stocks, a compromise between high growth rate and sporulation and a temperature at which the medium did not dry out too quickly. Chaxamycin production was also assessed at the same three temperatures in modified ISP2 production medium and shown to be very low at 37°C and 43°C compared to 30°C (see Fig. S3 in the supplemental material); consequently, all studies of chaxamycin production were performed at 30°C.

Before establishing techniques for the genetic manipulation of S. leeuwenhoekii, we first tested the susceptibility of the strain to a range of antibiotics commonly used with streptomycetes (16). Growth was prevented at concentrations of >1.0 μg/ml kanamycin, >0.5 μg/ml apramycin, >2.0 μg/ml hygromycin B, and >2.5 μg/ml thiostrepton; S. leeuwenhoekii was not sensitive to phosphomycin up to 50 μg/ml, while growth was retarded in the presence of nalidixic acid at >25 μg/ml (see Fig. S4 in the supplemental material).

Many plasmids used to manipulate streptomycetes genetically utilize the attachment sites of temperate phages (16). Putative bacterial attachment (*attB*) sites for the phiC31 and phiBT1 actinophages were identified in the chromosome of S. leeuwenhoekii (European Nucleotide Archive accession number PRJEB8583 [19]) by BLAST searches using the sequences of the corresponding sites from S. coelicolor (35, 36) (see Table S1 in the supplemental material). In both cases, the putative *attB* sites were located in homologues of the genes in which they reside in S. coelicolor (see Table S2 in the supplemental material).

Conditions for transferring DNA into S. leeuwenhoekii by conjugation were established using pSET152, which integrates in the phiC31 *attB* and carries the apramycin resistance gene *aac*(*3*)*IV*, and pIJ10257, which integrates at the phiBT1 *attB* and carries the hygromycin B resistance gene *hyg*; conjugations were plated on SFM agar supplemented with 10 mM MgCl_2_ and 10 mM CaCl_2_.

The most common constitutive promoter used for gene expression in Streptomyces is ermE*p (37). To test its usability in S. leeuwenhoekii, we introduced pGUS (a pSET152 derivative with the *gusA* reporter gene [38]) and pIJ10740 (a pGUS derivative with ermE*p driving the transcription of *gusA* [Morgan Feeney, personal communication]); *gusA* expression (assessed as described previously [39]) was readily observed in the pIJ10740 exconjugants but not in the pGUS exconjugants or in the S. leeuwenhoekii wild-type strain, confirming the utility of ermE*p in this species.

pGM1190 is a self-replicative, temperature-sensitive plasmid (reported to be nonreplicative at over 34°C) used for gene replacement in Streptomyces (40). We readily obtained S. leeuwenhoekii exconjugants with pGM1190 and verified plasmid loss after cultivation at 34°C, 37°C, and 43°C (see Fig. S5 in the supplemental material). Since we observed some persistence of pGM1190 at 34°C, 37°C was used to cure the plasmid.

### Bioinformatic identification of the chaxamycin biosynthesis gene cluster.

Bioinformatic analysis of the genome sequence of S. leeuwenhoekii identified a contiguous stretch of DNA that contained many of the genes predicted to be required for chaxamycin biosynthesis, including those needed for AHBA biosynthesis (i.e., the previously sequenced AHBA synthase gene [GenBank accession number FR839674.1]) and a complete set of appropriate PKS modules, including a loading module with similarity to those of other ansamycin-type PKSs such as ansamitocin (DoBISCUIT accession code Ansam_00270), geldanamycin (Gelda2_00080), herbimycin A (Herb_00170), rifamycin (Rifam_00210), and rubradirin (Rubra_00070).

**FIG 2:**
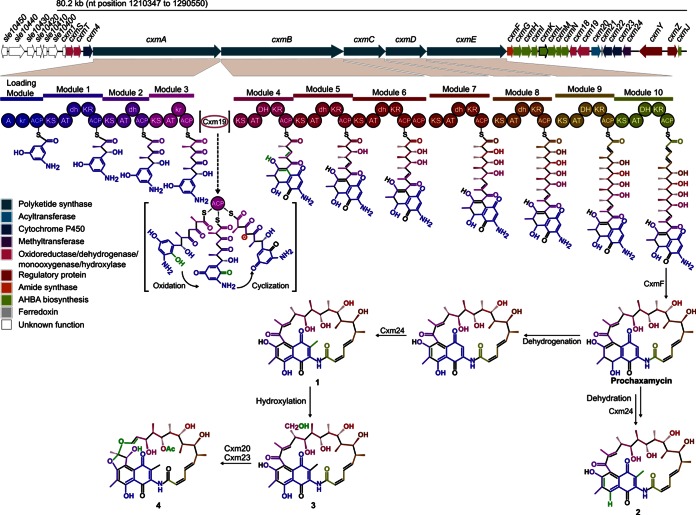
Top, organization of the chaxamycin gene cluster (*cxm*) of S. leeuwenhoekii. The proposed function of each gene is listed in Table 2. Bottom, proposed pathway for chaxamycin biosynthesis (based on that for rifamycin). The modules encoded in the polyketide synthase genes and their respective domains are as follows: A, adenylation; KS, ketosynthase; AT, acyltransferase; KR, ketoreductase; DH, dehydratase; ACP, acyl carrier protein. The domains in lower case should be inactive. Chaxamycins: 1, chaxamycin A; 2, chaxamycin B; 3, chaxamycin C; 4, chaxamycin D.

The putative limits of the gene cluster were identified by comparison with gene clusters encoding other ansamycin-like polyketides, including rifamycin (the *rif* cluster) from A. mediterranei S699 (AF040570.3), naphthomycin from Streptomyces sp. strain CS (GQ452266.1), and saliniketal from Salinispora arenicola CNS-205 (CP000850.1). The right end of the chaxamycin gene cluster (as shown in Fig. 2 and in Fig. S6 in the supplemental material) was defined by the presence of a gene, *cxmJ*, homologous to *rifJ*, encoding a putative aminodehydroquinate (amino-DHQ) dehydratase involved in the biosynthesis of AHBA (catalyzing the conversion of 5-deoxy-5-aminodehydroquinic acid to 5-deoxy-5-aminodehydroshikimic acid [amino-DHS] [14]); surprisingly, *cxmJ* is located some distance from the other genes required for AHBA biosynthesis, as also noted for the *cxmJ* homologues in the other three gene clusters (13).

The left end of the chaxamycin gene cluster (as shown in Fig. 2) could not be readily predicted, although from comparisons with the other ansamycin-like gene clusters, we initially speculated that *cxmS*, encoding a putative NADH-dependent dehydrogenase homologous to the *rifS* product, would be the likely limit of the cluster. At 8.7 kb upstream of *cxmS* (to the left of *cxmS* in Fig. 2), we found a conserved region of 11 genes (*sle10460* to *sle10560*) including a putative hopanoid biosynthesis gene cluster that is also present in *S. scabies* 87.22 (*scab_12881* to *scab_13001*, excluding *scab_12921* [41]) and S. coelicolor (*sco6759* to -*6771*, except *sco6761* [42]), as well as in many other streptomycetes. Between *cxmS* and the hopanoid biosynthesis gene cluster there are seven genes, four of which encode highly conserved proteins with homologues in S. coelicolor: Sle10450, Sle10430, Sle10420, and Sle10400 are homologous to Sco6772, Sco6773, Sco6774, and Sco6776, respectively. Two of the remaining genes (*sle10440* and *sle10410*) encode highly conserved proteins that have no close homologues in S. coelicolor. The gene immediately upstream of *cxmS*, *sle10390*, encodes a hypothetical protein without clear homologues in the databases; since the 3′ end of the coding sequence of *sle10390* overlaps with *cxmS* and there is a 720-bp intergenic region between *sle10390* and the gene immediately upstream, *sle10400*, it is conceivable that *sle10390* is part of the chaxamycin biosynthesis gene cluster, and thus this gene was assigned as the left limit of the cluster and named *cxm1* (Fig. 2 and Table 4).

**TABLE 4:** Proposed chaxamycin biosynthesis gene cluster and gene functions

*sle* no.	*cxm* name^*a*^	No. of amino acids	Rifamycin homologue (mutant phenotype)^*b*^	Proposed function in chaxamycin biosynthesis^*c*^
*sle10390*	*cxm1*	138		Small hypothetical protein
*sle10380*	*cxmS*	327	*rifS*	NADH-dependent oxidoreductase
*sle10370*	*cxmT*	323	*rifT*	NADH-dependent dehydrogenase
*sle10360*	*cxm4*	397	*orf0*	Cytochrome P450
*sle10350*	*cxmA*	5616	*rifA*	Polyketide synthase (module 0 [M0], A_AHBA_-KR-ACP; M1, KS-AT_mmal_-dh-KR-ACP; M2, KS-AT_mal_-DH-ACP; M3, KS-AT_mmal_-KR-ACP)
*sle10340*	*cxmB*	5363	*rifB*	Polyketide synthase (M4, KS-AT_mmal_-dh-KR-ACP; M5, KS-AT_mmal_-dh-KR-ACP; M6, KS-AT_mmal_-dh-KR-ACP-ACP)
*sle10330*	*cxmC*	1820	*rifC*	Polyketide synthase (M7, KS-AT_mmal_-dh-KR-ACP)
*sle10320*	*cxmD*	1773	*rifD*	Polyketide synthase (M8, KS-AT_mmal_-DH-KR-ACP)
*sle10300*	*cxmE*	3488	*rifE*	Polyketide synthase (M9, KS-AT_mal_-DH-KR-ACP; M10, KS-AT_mal_-DH-KR-ACP)
*sle10290*	*cxmF*	269	*rifF* (−)^*e*^	Polyketide release and ansa-ring formation: amide synthase
*sle10280*	*cxmG*	368	*rifG*^*d*^	AHBA synthesis, aminodehydroquinate synthase
*sle10270*	*cxmH*	406	*rifH*^*d*^	AHBA synthesis, amino-DAHP synthase
*sle10260*	*cxmI*	268	*rifI*^*d*^	AHBA synthesis, aminoquinate dehydrogenase
*sle10250*	*cxmK*	386	*rifK*^*d*^	AHBA synthesis, AHBA synthase
*sle10240*	*cxmL*	358	*rifL*^*d*^	AHBA synthesis, oxidoreductase
*sle10230*	*cxmM*	232	*rifM*^*d*^	AHBA synthesis, phosphatase
*sle10220*	*cxmN*	307	*rifN*^*d*^	AHBA synthesis, kanosamine kinase
*sle10210*	*cxm18*	295	*orf11* (+)^*f*^	Flavin-dependent oxidoreductase
*sle10200*	*cxm19*	533	*orf19* (−)^*f*^	Naphthalene ring formation, FDA-dependent-monooxygenase and 3-(3-hydroxylphenyl) propionate hydroxylase
*sle10190*	*cxm20*	402	*orf20*	Tailoring, *O*-acyltransferase (chaxamycin D)
*sle10180*	*cxm21*	63		Ferredoxin
*sle10170*	*cxm22*	393	*orf4* (+)^*f*^	Cytochrome P450 monooxygenase
*sle10160*	*cxm23*	418	*orf5* (W)^*f*^, *orf13* (+)^*f*^	Tailoring, cytochrome P450 monooxygenase (hydroxyfuran of chaxamycin D)
*sle10150*	*cxm24*	355		Tailoring: methyltransferase, *S*-adenosylmethionine dependent (not homologue of *orf14*)
*sle10120*	*cxmY*	433		Transcriptional regulator (C-terminal DNA-binding domain found in the NarL/FixJ response regulator family)
*sle10110*	*cxmZ*	246		Transcriptional regulator, atypical response regulator of OmpR/PhoB family
*sle10100*	*cxmJ*	168	*rifJ*^*d*^	AHBA synthesis, aminodehydroquinate dehydratase

a Gene names have been assigned as far as possible according to predicted functional homology to the rifamycin gene cluster (see also Table S3 in the supplemental material).

b Rifamycin gene cluster from A. mediterranei S699 (GenBank accession no. AF040570.3). Mutant phenotypes: +, similar production to parent strains; −, production abolished; W, loss of rifamycin B production and accumulation of rifamycin W.

c Based on Pfam motif search and homology to rifamycin biosynthesis proteins. PKS domains (found with antiSMASH [47] and NCBI-CDD [72]): A, adenylation; ACP, acyl carrier protein; AT, acyltransferase; DH, dehydratase (lowercase indicates that it should be inactive); KR, ketoreductase; KS, ketosynthase. The specificity predicted for each AT domain is shown as a subscript: AHBA, 3-amino-5-hydroxybenzoic acid; mal, malonyl-CoA; mmal, methylmalonyl-CoA.

d Genes required for AHBA biosynthesis (43).

e According to reference 56.

f Mutant accumulates linear polyketide intermediates (59).

The chaxamycin biosynthesis gene cluster of S. leeuwenhoekii, as defined above, spans a region of 80.2 kb, from nt 1210347 to 1290550 of the published genome sequence (19), and contains 27 genes. The core PKS and AHBA biosynthesis genes show very similar organization, and the encoded proteins high sequence identity, to the rifamycin biosynthesis gene cluster of A. mediterranei S699 (GenBank accession no. AF040570.3) (see Fig. S6 in the supplemental material). Consequently, gene names and predicted functions were assigned based on those defined in the rifamycin gene cluster (Fig. 2 and Table 4).

### Heterologous expression of the chaxamycin biosynthesis gene cluster.

One clone, pIJ12853, from a PAC library of S. leeuwenhoekii genomic DNA was identified by PCR and confirmed by sequencing to contain the proposed chaxamycin biosynthesis gene cluster (see Materials and Methods). pIJ12853 was mobilized into S. coelicolor M1152, resulting in integration of the gene cluster at the chromosomal phiC31 *attB* site, yielding strain M1650.

**FIG 3:**
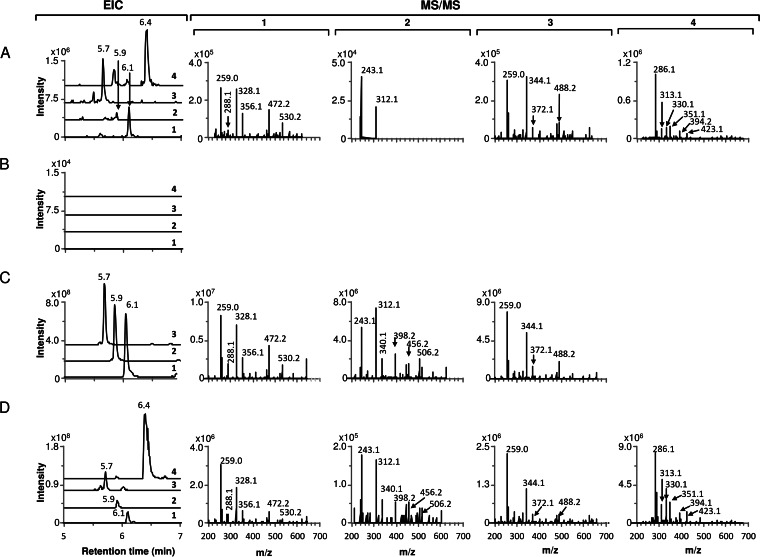
Heterologous production of chaxamycins A to D in S. coelicolor M1152. Extracted ion chromatogram (EIC) and MS/MS fragmentation patterns are shown for each chaxamycin species detected. (A) S. coelicolor M1650 (M1152 containing pIJ12853); (B) S. coelicolor M1152 (negative control); (C) chaxamycin A, B, and C standards; (D) S. leeuwenhoekii. Chaxamycin A (compound 1), *m/z* (M − H)^−^ 638.29; chaxamycin B (compound 2), *m/z* (M − H)^−^ 622.29; chaxamycin C (compound 3), *m/z* (M − H)^−^ 654.29; chaxamycin D (compound 4), *m/z* (M − H)^−^ 682.29.

M1650 was cultivated in R3 liquid medium, and culture supernatants and extracts of mycelial samples harvested at different times were analyzed by LC-MS, in parallel with chaxamycin standards and samples from S. leeuwenhoekii grown in modified ISP2 liquid medium. The expected molecular ions for chaxamycins A to D (compounds 1 to 4) were readily found in samples from M1650 and with retention times (6.1, 5.9, 5.7, and 6.4 min, respectively) similar to those for the purified standards and the samples from S. leeuwenhoekii (Fig. 3). In addition, the MS/MS fragmentation patterns of the ions detected for compounds 1, 3, and 4 in the M1650 samples matched those detected from the natural producer, while those for compounds 1 and 3 also matched the fragmentation patterns obtained from the standards; production of compound 2 in M1650 was very low and did not provide clear fragmentation data (Fig. 3B). These results demonstrated that the proposed gene cluster does indeed encode chaxamycin biosynthesis.

### Deletion of the AHBA synthase gene (*cxmK*) and chemical complementation.

AHBA is the starter unit for the synthesis of the polyketide backbone of chaxamycin. *cxmK* (Table 4) encodes a putative AHBA synthase that catalyzes both the transamination of UDP-3-keto-α-d-glucose at an early stage of the pathway and the final aromatization of amino-DHS to AHBA (13). Only one copy of this gene was found in the genome sequence of S. leeuwenhoekii, directly downstream of the PKS genes. *cxmK* was deleted and replaced with the kanamycin resistance gene *neo* by homologous recombination (see Materials and Methods and Fig. S7 in the supplemental material) to generate S. leeuwenhoekii M1653 (Δ*cxmK*::*neo*).

**FIG 4:**
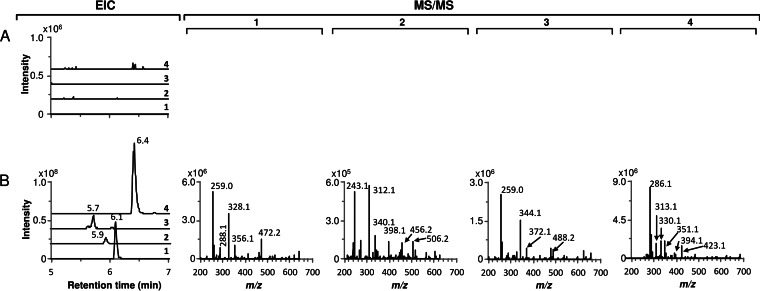
Chemical complementation of S. leeuwenhoekii M1653 (Δ*cxmK*::*neo*) with 3-amino-5-hydroxybenzoic acid (AHBA). Extracted ion chromatogram (EIC) and MS/MS fragmentation patterns are shown for each chaxamycin species detected. (A) S. leeuwenhoekii M1653; (B) S. leeuwenhoekii M1653 supplemented with 0.36 mM AHBA. Chaxamycin A (compound 1), *m/z* (M − H)^−^ 638.29; chaxamycin B (compound 2), *m/z* (M − H)^−^ 622.29; chaxamycin C (compound 3), *m/z* (M − H)^−^ 654.29; chaxamycin D (compound 4), *m/z* (M − H)^−^ 682.29.

M1653 was cultivated in parallel with wild-type S. leeuwenhoekii in chaxamycin production medium; LC-MS analysis of samples of culture supernatants harvested at different times after inoculation showed that biosynthesis of all of the chaxamycin species had been abolished in the Δ*cxmK* mutant M1653 (Fig. 4A). To confirm that the lack of production was due solely to the deletion of *cxmK*, we attempted to complement the mutant by expression in *trans* of a wild-type copy of *cxmK* cloned in the expression vector pIJ10257. The resulting plasmid, pIJ12852, was introduced into M1653 but failed to restore chaxamycin production, presumably reflecting a polar effect of the insertion of *neo* on the expression of other downstream AHBA biosynthesis genes. We then succeeded in complementing the mutant chemically, restoring chaxamycin production (Fig. 4B) by supplementing the culture medium with commercially available AHBA; after testing several concentrations and times of addition, optimal results were obtained when adding AHBA to 0.36 mM at 24 h after inoculation, presumably coinciding with production of the chaxamycin biosynthesis enzymes and minimizing AHBA degradation and/or catabolism. These feeding experiments confirmed a role for *cxmK* in AHBA synthesis and provided further evidence that the identified gene cluster does indeed encode chaxamycin biosynthesis. It also suggests that mutasynthesis, and the feeding of AHBA analogues, may be a viable approach for the generation of unnatural chaxamycin variants.

## DISCUSSION

### Functions of the genes present in the chaxamycin biosynthesis gene cluster and a proposed biosynthetic pathway.

We have unequivocally identified, cloned, genetically manipulated, and heterologously expressed the chaxamycin biosynthesis gene cluster from S. leeuwenhoekii. The chemical structures of the chaxamycins and the rifamycins share many similarities, and therefore it is not surprising that the biosynthesis gene clusters are also very similar in organization. Based on knowledge of rifamycin biosynthesis, we can now propose a biosynthetic pathway for chaxamycin.

The chaxamycin biosynthesis gene cluster (Fig. 2 and Table 4) contains homologues (*cxmG* to -*N* and *cxmJ*) of eight of the genes involved in AHBA biosynthesis in the rifamycin gene cluster of A. mediterranei S699 (*rifG* to -*N* and *rifJ*; like *rifJ*, *cxmJ* is not clustered with the other genes involved in AHBA synthesis). They show the same organization as the *rif* genes, and their products show high levels of amino acid sequence identity (68 to 86%). Therefore, we propose that biosynthesis of the chaxamycin PKS starting unit (AHBA) occurs as in rifamycin biosynthesis in A. mediterranei S699 (see Fig. S8 in the supplemental material) (13, 14, 43).

In addition to CxmG to -N and CxmJ, a transketolase is also required for AHBA biosynthesis to catalyze the conversion of 3-amino-3-deoxy-d-fructose-6-phosphate to imino-erythrose-4-phosphate (14). Indeed, in Salinispora arenicola CNS-205, deletion of *sare_1272* and *sare_1273*, which encode a two-subunit transketolase, from the shared rifamycin-saliniketal biosynthesis gene cluster abolished the production of both compounds (44). While the proposed chaxamycin biosynthesis gene cluster does not appear to possess such a function, there are two genes (*sle09700* and *sle09710*) located 47.4 kb to the right of it, contained within the insert of pIJ12853, that encode homologues of transketolase subunits from A. mediterranei (encoded by *rif15A* and *rif15B*, respectively [45]) that might fulfil this role. Other lines of evidence suggest that such a function can be provided by genes outside the biosynthesis gene cluster. In A. mediterranei, deletion of *rif15A* and *rif15B* (45), homologues of *sare_1272* and *sare_1273*, respectively, led to the accumulation of rifamycins SV and S, indicating that AHBA was still being produced in the mutant strain. In addition, the heterologous production of AHBA in S. coelicolor after cloning the AHBA genes *rifG* to -*N* and *rifJ* suggests that the transketolase step can be performed by an alternative enzyme encoded elsewhere in the genome (14, 43).

CxmI is homologous to RifI, an aminoquinate (amino-Q) dehydratase that catalyzes the conversion of amino-DHQ to amino-Q, with no obvious role in AHBA biosynthesis. Deletion of *rifI* in A. mediterranei led to the accumulation of 20 to 25% more AHBA, and a salvage function has been proposed for the enzyme (43, 46).

The type I PKS, responsible for the assembly of the chaxamycin polyketide backbone, is encoded by five genes, *cxmA* to -*E*. Bioinformatic analysis of their protein sequences was performed with antiSMASH (47), which uses two methods to predict the substrate specificity of the loading and AT domains (48, 49). antiSMASH predicted a loading module with specificity for AHBA, followed by a module selective for methylmalonyl-CoA, one for malonyl-CoA, six more for methylmalonyl-CoA, and finally two more for malonyl-CoA, which is entirely consistent and colinear with the reactions required to produce chaxamycin A (compound 1) (Fig. 2 and Table 4).

The loading module of CxmA contains an adenylation domain with 79% sequence identity to the adenylation domain of RifA in A. mediterranei, which catalyzes the reaction between a benzoate molecule and ATP to produce an activated aryl-AMP species (50, 51). The CxmA adenylation domain contains the AMP-binding and ATPase motifs, suggesting that it could function in a similar fashion to its homologue from A. mediterranei (see Fig. S9 in the supplemental material).

The chemical structure of compound 1 suggests that the dehydratase (DH) domains from modules 1, 2, 5, 6, 7, and 8 should be inactive. Active DH domains possess two conserved motifs, ^2409^HXXXGXXXXP and ^2571^D(A/V)(V/A)(A/L)(Q/H), where H2409 and D2571 (the numbers refer to amino acid positions in the DH4 domain of the erythromycin PKS of Saccharopolyspora erythraea) comprise the catalytic dyad of the dehydratase (52, 53). DH domains from modules 4, 9, and 10 of the chaxamycin PKS, predicted to be functional, all contain the second conserved motif DAALH and the conserved histidine residue in the motif HXXXGXXXXP (although the DH domain from module 9 has proline replaced by alanine). DH domains from modules 1 and 2, predicted to be nonfunctional, do not have any of the conserved motifs. DH domains from modules 5 and 8, also predicted to be nonfunctional, are shorter than the rest of the DH domains and do not contain the motif D(A/V)(V/A)(A/L)(Q/H); thus, despite having the conserved H residue in the active site, they lack the catalytic dyad. Surprisingly, the DH domains of modules 6 and 7 contain both active-site motifs, and we presume that there are other mutations that result in their inactivity (see Fig. S10 in the supplemental material).

The KR domain of module 3 lacks the active-site tyrosine residue (54) and the NADPH-binding motifs (55); the KR domain present in the loading module is shorter and lacks both of sequence features mentioned above. Therefore, these KR domains are predicted to be inactive, again in agreement with the structure of chaxamycin. The remaining KR domains have the tyrosine residue and the conserved motifs for NADPH binding (see Fig. S11 in the supplemental material) and are predicted to be active, consistent with the structure of the polyketide (Fig. 2).

The chaxamycin PKS shows a high level of similarity to that involved in rifamycin biosynthesis in A. mediterranei S699 but contains two additional nonfunctional domains (an extra KR domain in the loading module and an extra DH domain in module 2) as well as, unusually, an apparently functional additional acyl carrier protein (ACP) domain in module 6. As for the rifamycin PKS, the chaxamycin PKS lacks a type I thioesterase domain at the end of module 10, and a putative *N*-acetyltransferase/amide synthase is encoded by *cxmF*, the likely orthologue of *rifF*, and is similarly located just downstream of the last PKS gene (*cxmE*) (Fig. 2). Therefore, we propose that CxmF catalyzes the formation of the internal amide bond between AHBA and the last extender molecule of the polyketide chain to close the macrolactam ring, as in rifamycin biosynthesis (56), thus releasing the PKS product, which we have called prochaxamycin (Fig. 2).

In rifamycin biosynthesis, a type II thioesterase, RifR, is thought to function in the release of aberrant intermediates from the PKS, thus restoring functionality for a new cycle (57). However, it is not essential, and the PKS itself may be capable of such activity (58). Interestingly, we identified a nonfunctional *rifR* homologue located between *cxmY* and *cxm24* that contained three frameshift mutations (the frameshift-corrected amino acid sequence shows end-to-end similarity and 64% identity to RifR); possible errors in the PacBio-Illumina assembled DNA sequence were ruled out by PCR amplification and Sanger sequencing (see Materials and Methods). Whether an alternative type II thioesterase encoded outside the *cxm* gene cluster plays an editing role for the chaxamycin polyketide synthase remains to be determined.

We identified eight genes encoding putative tailoring activities homologous to those found in the rifamycin gene cluster of A. mediterranei. In rifamycin biosynthesis, the naphthalene ring is formed during the extension of the polyketide backbone by the enzyme 3-(3-hydroxylphenyl)propionate hydroxylase encoded by *orf19* (59); a homologous gene, *cxm19*, is also present in the chaxamycin gene cluster, and the encoded protein is predicted to catalyze the formation of the naphthalene ring of chaxamycin.

A putative methyltransferase is encoded by *cxm24*, but it shows little similarity (25% identity over 87 residues of the 355-amino-acid Cxm24) to the C-27 *O*-methyltransferase (Rif-Orf14) involved in rifamycin biosynthesis. This is the only putative methyltransferase encoded by the chaxamycin gene cluster, and although Pfam predicts that it is an *O*-methyltransferase, we speculate that it is responsible for C methylation of the C-3 of AHBA in the naphthalene ring.

An *O*-acetyltransferase encoded by *orf20* catalyzes the O acetylation of C-25 during rifamycin biosynthesis (60). *cxm20*, encoding a homologous protein with 63% identity to Rif-Orf20, is thus proposed to catalyze the O acetylation of the hydroxyl group of C-25 in chaxamycin D (9). In rifamycin biosynthesis by A. mediterranei S699, a cytochrome P450 monooxygenase (CYP) encoded by *orf5* is required for the formation of the ketofuran moiety present in rifamycins SV and B but not in rifamycin W; a very similar CYP encoded by *orf13* seems to be nonessential for rifamycin B biosynthesis (59). We identified only one gene, *cxm23*, which encodes a putative CYP homologous to rifamycin Orf5 or Orf13 (68% and 72% amino acid sequence identity, respectively). This is similar to the rifamycin gene cluster from S. arenicola CNS-205, where only one encoded protein, Sare1259, is homologous to Orf5 and Orf13 from A. mediterranei S699 (with higher identity to Orf13 [79%] than to Orf5 [71%]). Sare1259 performs several oxidation reactions at different positions on the molecule (44) and is 77% identical to Cxm23; therefore, we propose that Cxm23 participates in the formation of the hydroxyfuran ring present in chaxamycin D but not in chaxamycin A, B, or C (Fig. 2).

### Transcriptional regulation.

Two genes encode putative transcriptional regulatory proteins, *cxmY* and *cxmZ*. Analysis of CxmY with Pfam (61) revealed a carboxy-terminal helix-turn-helix (HTH) DNA-binding domain (PF00196) typical of the NarL/FixJ family of response regulators (RRs) (62); the rest of the amino acid sequence shows no similarity to that protein family, but BLASTp analysis (21) revealed many homologues of putative transcriptional regulators in other actinomycetes, including Rif-Orf36 of the rifamycin gene cluster of A. mediterranei S699, with which it shows end-to-end similarity and 43% amino acid sequence identity. Similar analysis of CxmZ revealed a carboxy-terminal DNA-binding domain of the winged HTH family (Trans_reg_C family, PF00486) typical of the OmpR/PhoB family of RRs (62), and an amino-terminal RR receiver domain (Response_reg family, PF00072). CxmZ lacks the first of a pair of aspartates at positions 13 (serine) and 14 (aspartate) that determine the structure of the phosphorylation pocket in typical RRs, and it is thus unlikely to be phosphorylated by a cognate histidine sensory kinase (no such protein is encoded in the *cxm* gene cluster), supporting its classification as an atypical RR (see Fig. S12 in the supplemental material). CxmZ shows 41% amino acid sequence identity to JadR1, an atypical response regulator involved in the regulation of jadomycin biosynthesis in Streptomyces venezuelae (GenBank accession no. AAB36584.2). Although contiguous, *cxmY* and *cxmZ* are transcribed in the opposite direction with an intergenic region of over 600 bp that presumably contains the promoter sequences of both genes.

### Transport and immunity.

Surprisingly we did not find any gene(s) encoding a putative transport system within, or in close proximity to, the *cxm* gene cluster. The rifamycin biosynthesis gene cluster of A. mediterranei S699 encodes an efflux transporter (encoded by *rifP*) whose transcription is likely repressed by a TetR-like transcriptional regulator encoded by the gene immediately downstream (*rifQ*) (46, 63). Homologues of these two genes are absent from the chaxamycin gene cluster and from the rifamycin/saliniketal gene cluster of S. arenicola CNS-205, which also does not seem to encode a transport system (44). However, two RifP homologues are encoded elsewhere in the S. leeuwenhoekii genome: Sle37020 and Sle08130 (62% and 60% amino acid sequence identity to RifP, respectively). Neither of the corresponding genes is present in pIJ12853. S. coelicolor M145, from which strain M1152 was derived, also encodes a RifP homologue, Sco4024 (62% identity to RifP). Sco4024 is 78% identical to Sle37020 and 64% identical to Sle08130. All of these predicted proteins (as well as RifP) contain the major facilitator superfamily (MFS) domain and could be responsible for export of chaxamycin from their respective strains. A *rifP* homologue (*sare_2597*) also occurs in the genome of S. arenicola CNS-205, outside the rifamycin/saliniketal gene cluster.

Rifamycin binds to the β subunit (RpoB) of RNA polymerase, and in A. mediterranei a rifamycin-resistant form of the protein is encoded by an *rpoB* homologue located immediately downstream of *rifJ-orf36-orf37* (64). In S. leeuwenhoekii, *rpoB* (nt 3577583 and 3581068) lies 1.29 Mb away from the chaxamycin gene cluster (nt 1211049 to 1289829), and its product does not have the amino acid residues required for resistance to rifamycin at the expected positions, instead possessing the same amino acids as S. coelicolor M145 RpoB, which is sensitive to rifamycin (see Fig. S13 in the supplemental material). Thus, it seems unlikely that chaxamycin exerts its antibiotic activity by binding to RpoB.

This study has provided new insights into chaxamycin biosynthesis. We have succeeded in expressing the gene cluster heterologously, thus providing a convenient means for a more detailed study of the biosynthetic pathway and the enzymes involved. We have also shown that genetic manipulation of the natural producer is feasible, which could aid in the future engineering of the strain and the directed production of particular chaxamycin species.

## Supplementary Material

Supplemental material
