# Supplementary material for: Identification and Heterologous Expression of the Chaxamycin Biosynthesis Gene Cluster from Streptomyces leeuwenhoekii
Source: Appl Environ Microbiol. 2015 Aug 7;81(17):5820–31. doi: 10.1128/AEM.01039-15 (PMC4551226; doi:10.1128/AEM.01039-15)
Supplement: Supplemental material [file supp_81_17_5820__index.html]

Identification and Heterologous Expression of the Chaxamycin Biosynthesis Gene Cluster from Streptomyces leeuwenhoekii — Supplemental material 

# Identification and Heterologous Expression of the Chaxamycin Biosynthesis Gene Cluster from Streptomyces leeuwenhoekii

## Supplemental material

- Supplemental file 1 -

  Growth and sporulation (Fig. S1), growth at different temperatures (Fig. S2), chaxamycin production (Fig. S3), antibiotic susceptibility (Fig. S4), usability of the replicative temperature-sensitive vector pGM1190 in *S. leeuwenhoekii* (Fig. S5), comparison of the chaxamycin biosynthetic gene cluster from *Streptomyces leeuwenhoekii* with the saliniketal/rifamycin biosynthetic gene cluster from *Salinispora arenicola* CNS-205 (Fig. S6), methodology used to construct *S. leeuwenhoekii* M1653 (Δ*cxmK*::*neo*) (Fig. S7), proposed biosynthesis of 3-amino-5-hydroxybenzoic acid (AHBA) through the amino-shikimate pathway in *Streptomyces leeuwenhoekii* (Fig. S8), comparison of the amino acid sequence of the adenylation domain present in the loading module of the chaxamycin polyketide synthase (PKS) with other adenylation domains from PKSs of known specificity (Fig. S9), manually curated bioinformatic analysis of DH domains present in the chaxamycin PKS to assess dehydratase activity (Fig. S10), manually curated bioinformatic analysis of KR domains present in the chaxamycin PKS to assess reductase activity (Fig. S11), predicted secondary structure of CxmZ and evaluation of the presence of catalytically relevant residues (Fig. S12), alignment of RpoB sequences (Fig. S13), DNA sequences of phage integration sites (Table S1), genes in which phage integration sites are located (Table S2), and identity between proteins encoded by the chaxamycin and other ansamycin-type biosynthetic gene clusters (Table S3).

  PDF, 3.9M
