# Supplementary material for: Identification and Heterologous Expression of the Chaxamycin Biosynthesis Gene Cluster from Streptomyces leeuwenhoekii
Source: Appl Environ Microbiol. 2015 Aug 7;81(17):5820–31. doi: 10.1128/AEM.01039-15 (PMC4551226; doi:10.1128/AEM.01039-15)
Supplement: Supplemental material [file AEM.01039-15_zam999116510so1.pdf]

Supplemental Material

Identification and heterologous expression of the chaxamycin biosynthetic gene cluster from *Streptomyces leeuwenhoekii*

Jean Franco Castro,[a,b] Valeria Razmilic,[a,b] Juan Pablo Gomez-Escribano,[b] Barbara Andrews,[a] Juan A. Asenjo,[a] Mervyn J. Bibb[b]#

Centre for Biotechnology and Bioengineering (CeBiB), Department of Chemical Engineering and Biotechnology, Universidad de Chile, Santiago, Chile [a]; Department of Molecular Microbiology, John Innes Centre, Norwich Research Park, Norwich, United Kingdom [b]

Running head: Chaxamycin gene cluster of *S. leeuwenhoekii*

# Address correspondence to Mervyn J. Bibb, mervyn.bibb@jic.ac.uk

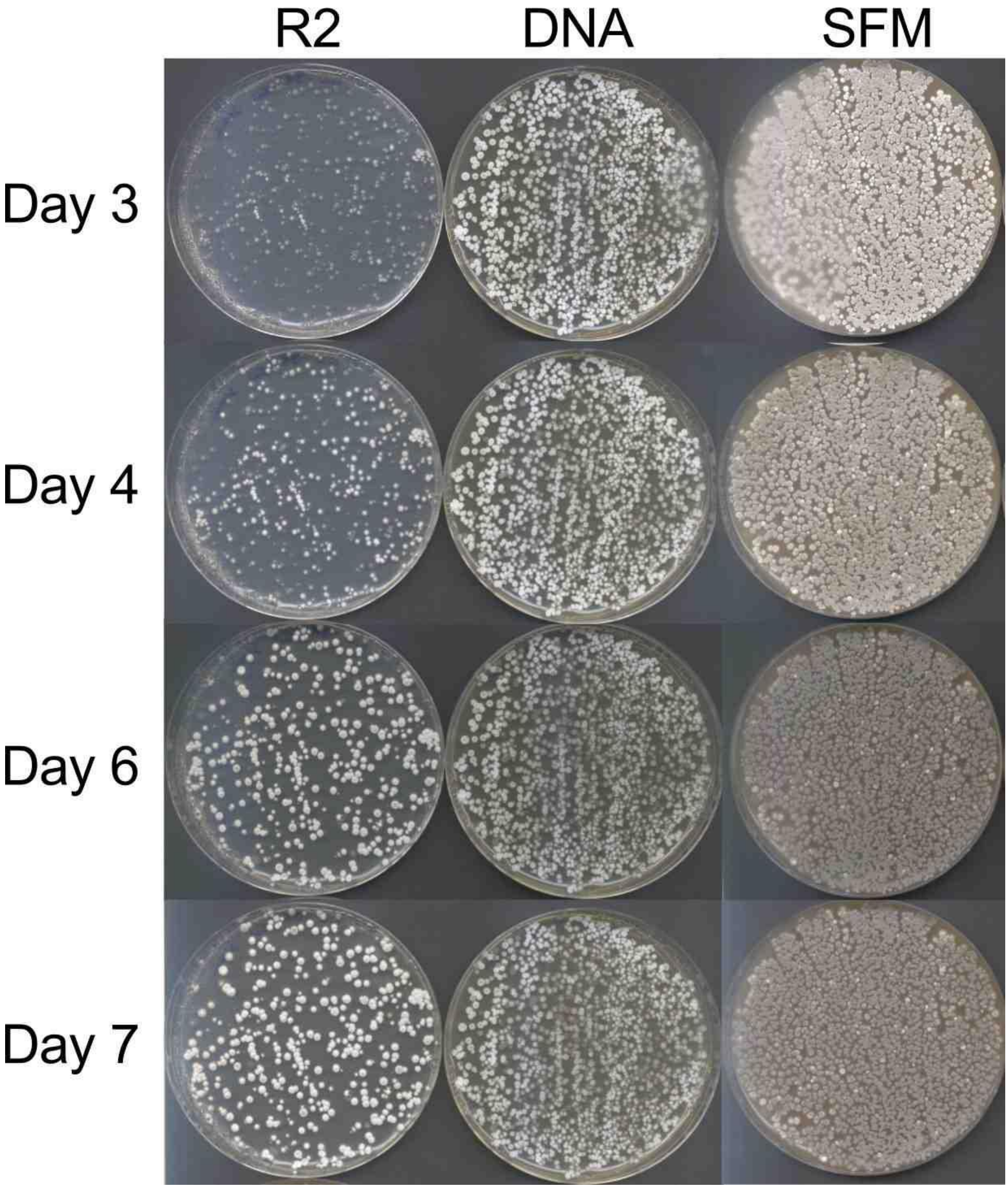

FIG S1 Evaluation of growth and sporulation of *S. leeuwenhoekii* on R2, DNA and SFM agar media at 30 °C. All media were supplemented with trace elements as for R2. Recipes from (1).

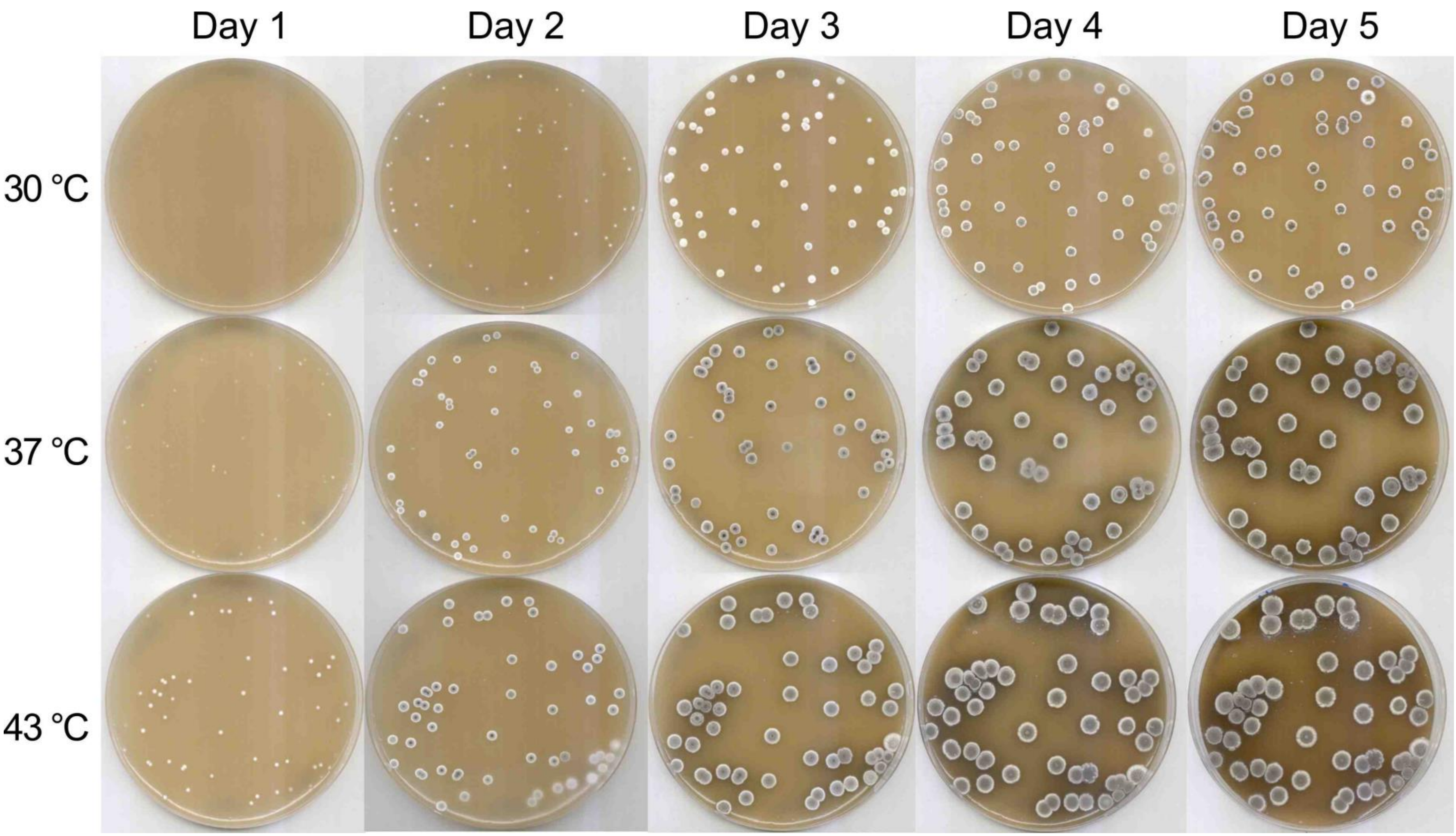

FIG S2 Evaluation of growth of *S. leeuwenhoekii* at different temperatures (30 °C, 37 °C and 43 °C) for 5 days on SFM agar. Fastest growth and sporulation were observed at 43 °C.

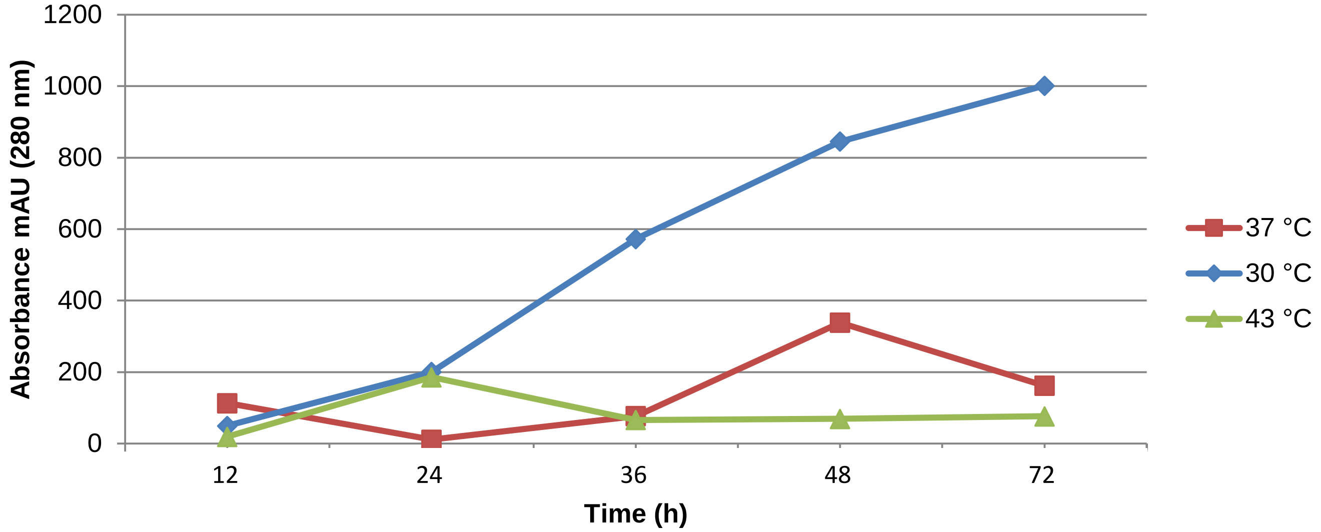

FIG S3 Evaluation of chaxamycin production by *S. leeuwenhoekii* in modified ISP2 liquid medium at different temperatures (30 °C, 37 °C and 43 °C). Samples were analysed by LC-MS as described in Materials and Methods; chromatograms were recorded by detection of absorbance at 280 nm (2); peaks corresponding to the different chaxamycin species were identified by their retention times compared with standards, and by mass spectrometry; chaxamycin production was estimated as the sum of the area of the peaks at 280 nm.

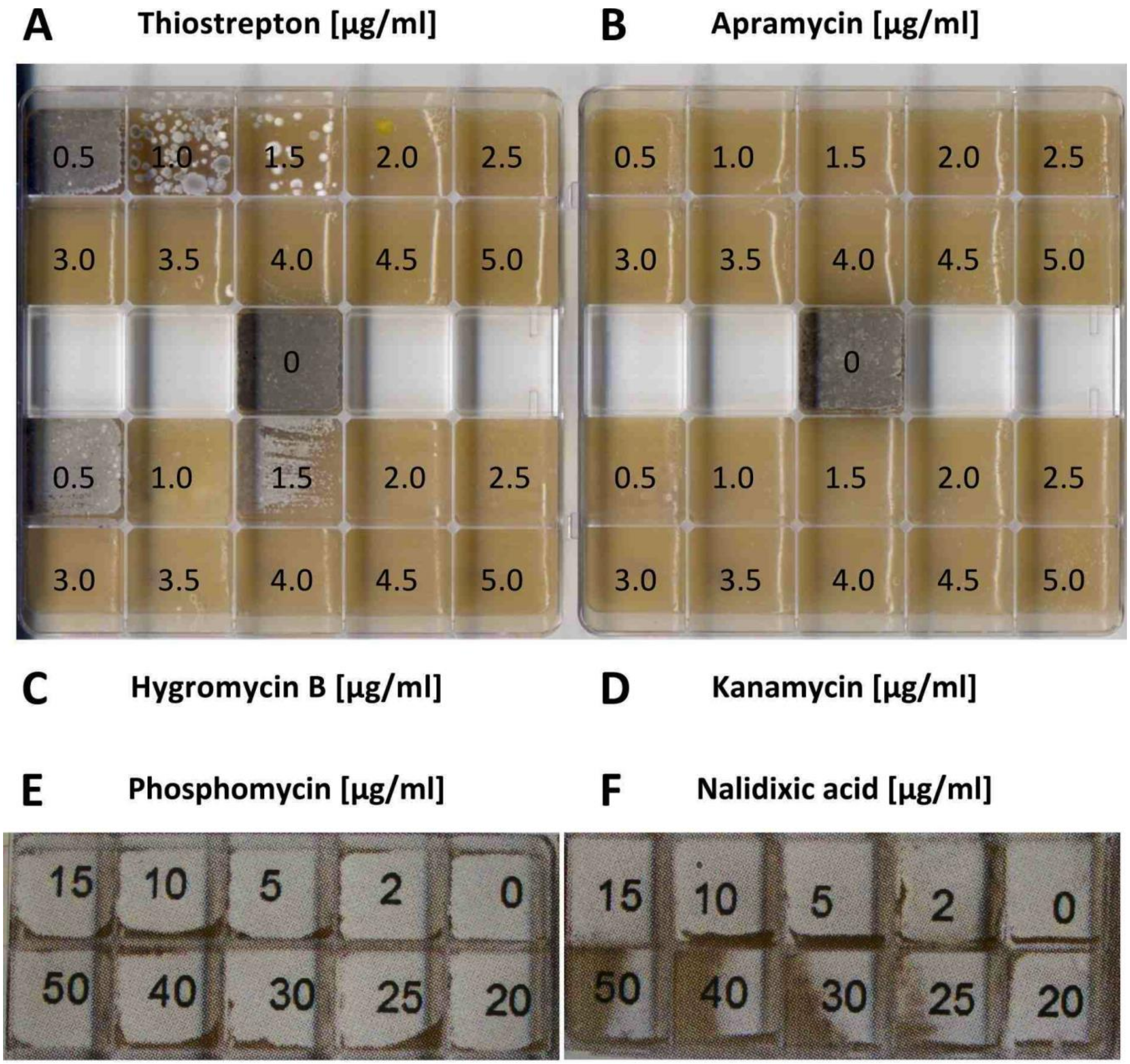

FIG S4 Evaluation of *S. leeuwenhoekii* susceptibility to antibiotics commonly used for selection during genetic manipulation in actinomycetes. To obtain agar medium homogeneously supplemented with the antibiotic, 2 ml Eppendorf tubes with 2 ml of SFM agar were kept in a heating block set at 50 °C and the required amount of antibiotic stock was added to each tube and homogenised by pipetting; the content of each tube was then transferred into a separate well of a square Petri dish (10 cm side, with 25 square wells). Each well was inoculated with 10^8 spores of *S. leeuwenhoekii*; plates were incubated at 30 °C for 7 days. Note: the well containing 1.0 microgram/ml hygromycin B failed to receive a spore inoculum; repetition of the experiment indicated complete susceptibility to2.0 microgram/ml hygromycin B.

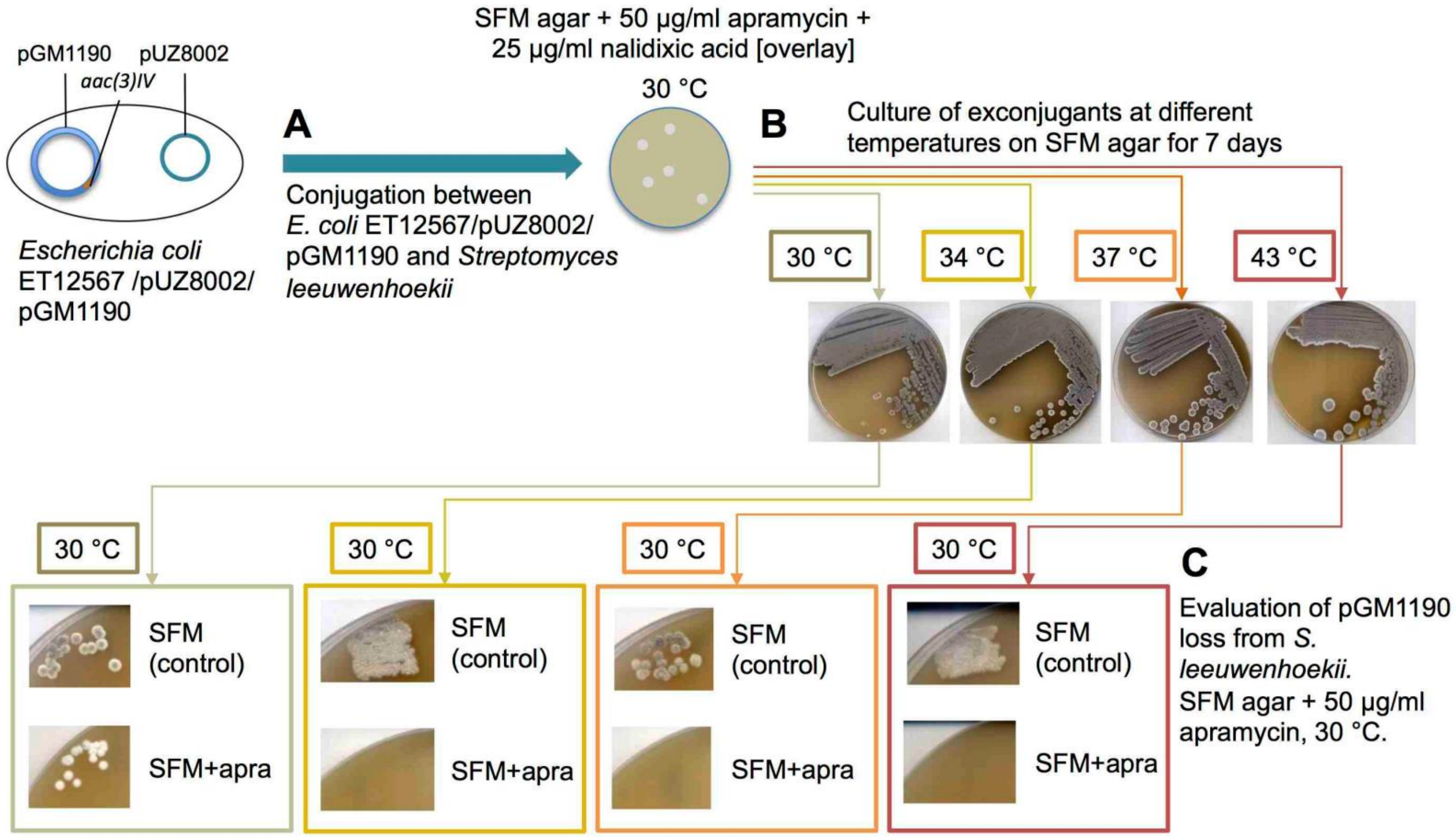

FIG S5 Evaluation of the usability of the replicative temperature-sensitive vector pGM1190 in *S. leeuwenhoekii*. (**A**) pGM1190 (which carries the apramycin resistance gene *aac(3)IV*) was mobilised into *S. leeuwenhoekii* by conjugation from *E. coli* ET12567/pUZ8002, and exconjugants were selected for apramycin resistance (50 µg/ml). (**B**) Spores from selected exconjugants were streaked in SFM agar without antibiotic and cultured at 30 °C, 34 °C, 37 °C or 43 °C. (**C**) Isolated colonies from each plate were replicated to both SFM and SFM + apramycin agar and cultured at 30 °C for 4 days. Clones from plates previously incubated at 37 °C or 43 °C did not grow in the presence of apramycin, indicating the loss of pGM1190; only a few clones from plates previously incubated at 34 °C grew in the presence of apramycin, indicating persistence of pGM1190 at a low frequency. Since *S. leeuwenhoekii* grows well at 37 °C we chose this temperature for all further experiments (see FIG S7).

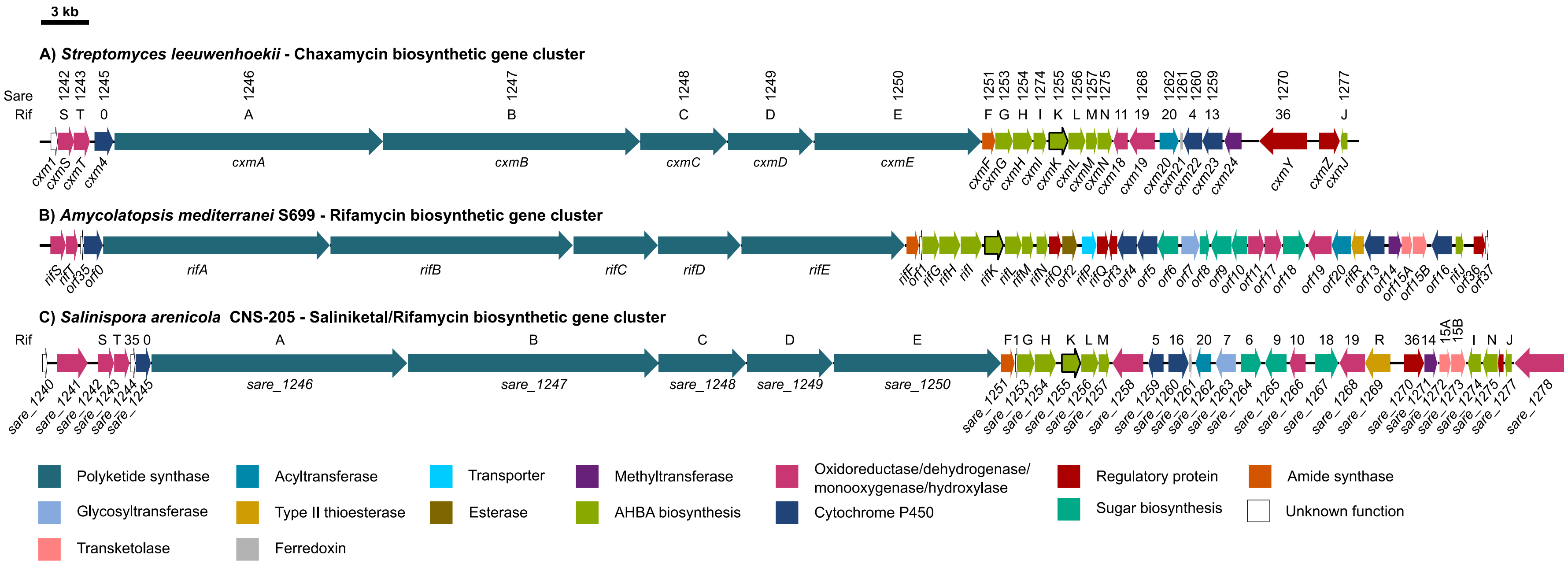

FIG S6 Comparison of the chaxamycin biosynthetic gene cluster (*cxm*) from *Streptomyces leeuwenhoekii* (**A**) with the saliniketal/rifamycin biosynthetic gene cluster (*sare*) from *Salinispora arenicola* CNS-205 (**B**; CP000850.1) and the rifamycin biosynthetic gene cluster (*rif*) from *Amycolatopsis mediterranei* S699 (**C**; AF040570.3).

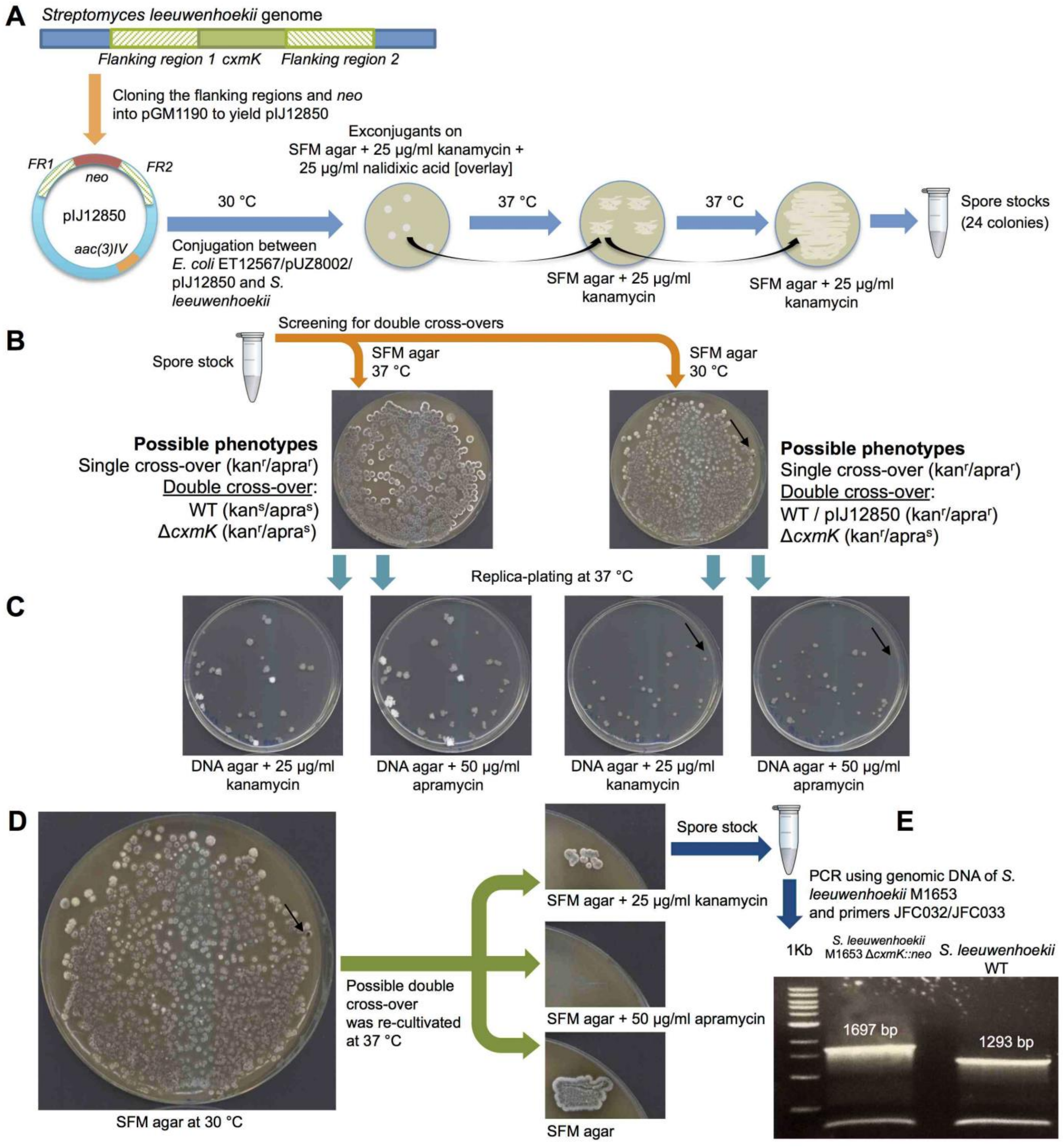

FIG S7 Methodology used to construct *S. leeuwenhoekii* M1653 (Δ*cxmK::neo*). (**A**) Flanking regions of the AHBA synthase gene (*cxmK*) and kanamycin resistance gene *neo* were cloned into pGM1190 (which carries the apramycin resistance gene *aac(3)IV*) to yield pIJ12850, which was then mobilised into *S. leeuwenhoekii* by conjugation from *E.*

coli ET12567/pUZ8002/pIJ12850 and the plates incubated at 30 °C (see Materials and Methods). Several kanamycin-resistant exconjugants were replicated on to SFM agar containing kanamycin and incubated at 37 °C, a temperature at which the vector cannot replicate and therefore all growing colonies must have integrated the resistance gene into the chromosome by, at least, a single cross-over recombination event. Spore stocks from 24 kanamycin-resistant exconjugants were prepared from plates incubated at 37 °C, and used for screening of double cross-over recombinants. (**B**) Spores were plated on SFM agar without antibiotics and incubated at either 30 or 37 °C. (**C**) After seven days, colonies were replica-plated sequentially on DNA agar medium supplemented with kanamycin and DNA agar with apramycin, and cultivated at 37 °C for one day. The different phenotypes were expected to be: $kan^r/apra^r$ (pIJ12850 still integrated in the chromosome, no second cross-over event); $kan^s/apra^s$ if a second cross-over event excised pIJ12850 from the chromosome followed by loss of the plasmid (and hence apramycin and kanamycin resistance) at 37 °C or $kan^r/apra^s$ if a second cross-over event excised the pIJ12850 backbone (losing the apramycin resistance gene) while at the same time replacing *cxmK* with *neo* leading to the mutant Δ*cxmK::neo*. (**D**) One $kan^r/apra^s$ colony (indicated by the arrow) was streaked sequentially on SFM agar supplemented with kanamycin, SFM agar supplemented with apramycin and SFM agar without antibiotic as control, and the $kan^r/apra^s$ phenotype expected for the Δ*cxmK::neo* mutant confirmed. (**E**) PCR analysis with primers JFC032/JFC033 confirmed that the chosen clone had the expected amplicon size for the replacement of *cxmK* with *neo*; the PCR product was confirmed by Sanger sequencing using the same primers. The resulting mutant strain was called *S. leeuwenhoekii* M1653 (Δ*cxmK::neo*).

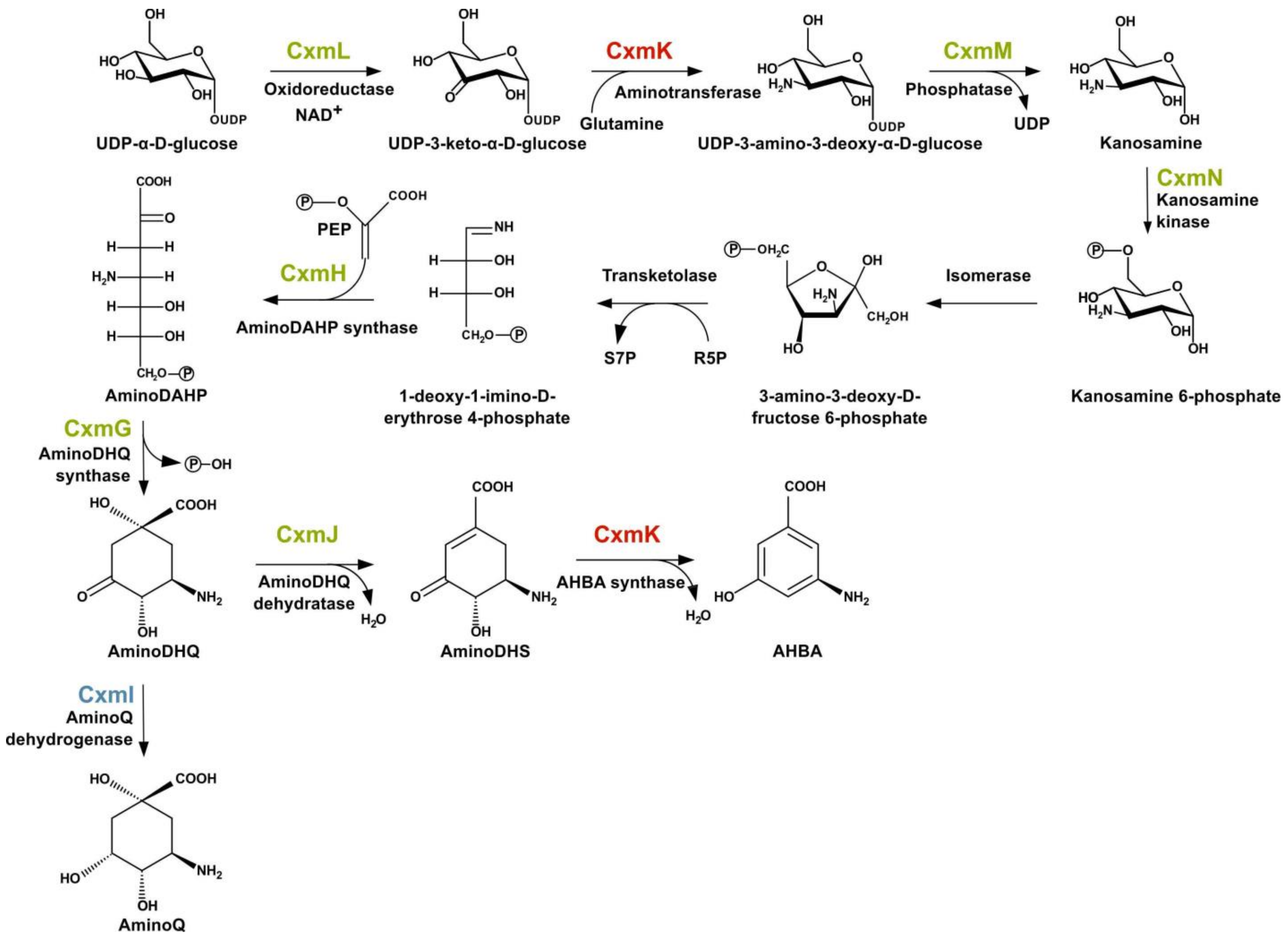

FIG S8 Proposed biosynthesis of 3-amino-5-hydroxybenzoic acid (AHBA) through the amino-shikimate pathway in *Streptomyces leeuwenhoekii*. This pathway was first studied in *Amycolatopsis mediterranei* and serves as a reference for the identification of homologous genes in *S. leeuwenhoekii* (3). The pathway begins with the conversion of UDP-α-D-glucose into UDP-3-keto-α-D-glucose by CxmL (homologue of RifL). This is then converted into UDP-3-amino-3-deoxy-α-D-glucose by AHBA synthase, CxmK (highlighted in red, top; homologue of RifK); the phosphatase, CxmM (homologue of RifM), uses UDP-3-amino-3-deoxy-α-D-glucose as substrate to produce kanosamine. Kanosamine is then converted into D-kanosamine 6-phosphate by the enzyme kanosamine kinase, CxmN (homologue of RifN). The subsequent steps in the pathway towards AHBA are performed by a transketolase, followed by an isomerase that yields 1-deoxy-1-imino-D-erythrose 4-phosphate. In other microorganisms, such as *S. coelicolor* and *Escherichia coli*, the transketolase activity can be performed by a housekeeping transketolase (4); in *Salinispora arenicola* CNS-205 it can be performed by a dedicated transketolase located within the rifamycin/saliniketal biosynthetic gene cluster (genes *Sare_1272* and *Sare_1273*) (5); in *A. mediterranei* S699 it is

predicted that the transketolase *rif15A* and *rif15B* (designated *AMED_0651* and *AMED_0652* in strain U32), also located within the rifamycin biosynthetic gene cluster, could perform this step (6). The chaxamycin biosynthetic gene cluster does not contain a transketolase homologue, so presumably a transketolase encoded elsewhere in the genome performs this step in *S. leeuwenhoekii*. 1-deoxy-1-imino-D-erythrose 4-phosphate is the substrate of the enzyme 3,4-dideoxy-4-amino-D-arabino-heptulosonate 7-phosphate (aminoDAHP) synthase, CxmH (homologue of RifH), which produces aminoDAHP. AminoDAHP is then converted into 5-deoxy-5-amino-3-dehydroquinate (aminoDHQ) by CxmG (homologue of RifG). CxmJ, an amino DHQ dehydratase (homologue of RifJ), converts aminoDHQ into 5-amino-5-deoxy-3-dehydroshikimate (aminoDHS), which is used by the AHBA synthase, CxmK (highlighted in red, bottom), to yield the final product AHBA. CxmI (homologue of RifI; highlighted in blue) is an amino quinate (aminoQ) dehydratase, which catalyses the conversion of aminoDHQ into aminoQ, suggesting a salvage function. Deletion of *rifI* in *A. mediterranei* led to an accumulation of 20–25% more AHBA (7).

UDP = uridine diphosphate; kanosamine = 3-amino-3-deoxy-D-glucose; R5P = D-ribose 5-phosphate; S7P = D-sedoheptose 7-phosphate; aminoDAHP = 3,4-dideoxy-4-amino-D-arabino-heptulosonate 7-phosphate; aminoDHQ = 5-deoxy-5-amino-3-dehydroquinate; aminoQ = amino quinate; aminoDHS = 5-amino-5-deoxy-3-dehydroshikimate; AHBA = 3-amino-5-hydroxybenzoic acid.

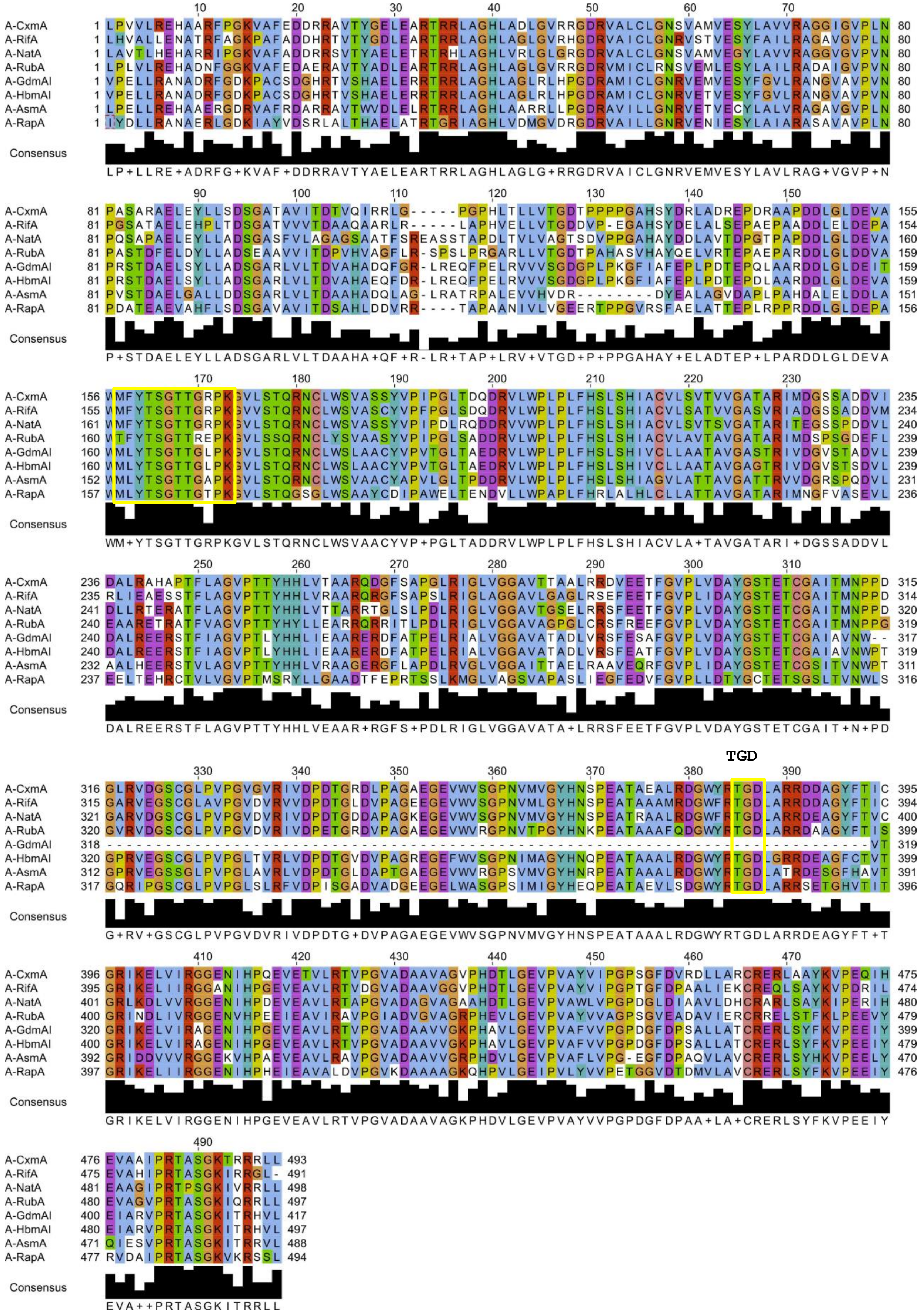
A-CxmA 1 LPVVLREHAARFPGKVAFEDDRRAVTYGELEARTRRLAGHLADLGVRRGDRVALCLGNSVAMVESYLAVVRAGGIGVPLN 80
A-RifA 1 LHVALLENATRFAGKPAFADDHRTVTYGDLEARTRRLAGHLAGLGVRHGDRVAICLGNRVSTVESYFAILRAGAVGVPLN 80
A-NatA 1 LAVTLHEHARRIPGKVAFADDRRSVTYAELETRTRHLAGHLVRLGLGRGDRVALCLGNSVAMVEGYLAVVRAGGVGVPVN 80
A-RubA 1 LPLVLREHADNFGGKVAFEDAERAVTYADLEARTRRLAGHLAGLGVRRGDRVMICLRNSVEMLESYLAILRADAIGVPVN 80
A-GdmAI 1 VPELLRANADRFGDKPACSDGHRTVSHAELERRTRRLAGHLAGLRLHPGDRAMICLGNRVEMVESYFGVLRANGVAVPVN 80
A-HbmAI 1 VPELLRANADRFGDKPACSDGHRTVSHAELERRTRRLAGHLAGLRLHPGDRAMICLGNRVETVESYFGVLRANGVAVPVN 80
A-AsmA 1 LPELLREHAAERGDRVAFRDARRAVTWVDLELRTRRLAGHLAARRLLPGDRAVILLGNRVETVECYLALVRAGAVGVPLN 80
A-RapA 1 IYDLLRANAERLGDKIAYVDSRLALTHAELATRTGRIAGHLVDMGVDRGDRVAILLGNRVENIESYLAIARASAVAVPLN 80
Consensus
LP+LLRE+ADRFG+KVAF+DDRRAVTYAELEARTRRLAGHLAGLG+RRGDRVAICLGNRVEMVESYLAVLRAG+VGVP+N
A-CxmA 81 PASARAELEYLLSDSGATAVITDTVQIRRLG-----PGPHLTLLVTGDTPPPPGAHSYDRLADREPDRAAPDDLGLDEVA 155
A-RifA 81 PGSATAELEHPLTDSGATVVVTDAAQAARLR-----LAPHVELLVTGDDVP-EGAHSYDELALSEPAEPAADDLELDEPA 154
A-NatA 81 PQSAPAELEYLLADSGASFVLAGAGSAATFSREASSTAPDLTVLVAGTSDVPPGAHAYDPLAVTDPGTPAPDDLGLDEVA 160
A-RubA 81 PASTDFELDYLLADSEAAVVITDPVHVAGFLR-SPSLPRGARLLVTGDTPAHASVHAYQELVRTEPAEPARDDLGLDDVA 159
A-GdmAI 81 PRSTDAELSYLLADSGARLVLTDVAHADQFGR-LREQFPELRVVVSGDGPLPKGFIAFEPLPDTEPQLAARDDLGLDEIT 159
A-HbmAI 81 PRSTDAELSYLLADSGARLVLTDVAHAEQFDR-LREQFPELRVVVSGDGPLPKGFIAFEPLPDTEPDLAARDDLGLDEVA 159
A-AsmA 81 PVSTDAELGALLDDSGARLVLTDAAHADQLAG-LRATRPALEVVHVDR--------DYEALAGVDAPLPAHDALELDDLA 151
A-RapA 81 PDATEAEVAHFLSDSGAVAVITDSAHLDDVRR----TAPAANIVLVGEERTPPGVRSFAELATTEPLRPPRDDLGLDEPA 156
Consensus
P+STDAELEYLLADSGARLVLTDAAHA+QF+R-LR+TAP+LRV+VTGD+P+PPGAHAY+ELADTEP+LPARDDLGLDEVA
A-CxmA 156 VMFYTSGTTGRPKGVLSTQRNCLWSVASSYVPIPGLTDQDRVLWPLPLFHSLSHIACVLSVTVVGATARIMDGSSADDVI 235
A-RifA 155 VMFYTSGTTGRPKGVVSTQRNCLWSVASCYVPFPGLSDQDRVLWPLPLFHSLSHIACVLSATVVGASVRIADGSSADDVM 234
A-NatA 161 VMFYTSGTTGRPKGVLSTQRNCLWSVASSYVPIPDLRQDDRVVWPLPLFHSLSHIACVLSVTSVGATARITEGSSPDDVL 240
A-RubA 160 VTFYTSGTTREPKGVLSSQRNCLYSVAASYVPIPGLSADDRVLWPLPLFHSLSHIACVLAVTAVGATARIMDSPSGDEFL 239
A-GdmAI 160 VMLYTSGTTGLPKGVLSTQRNCLWSLAACYVPVTGLTAEDRVLWPLPLFHSLSHIVCLLAATAVGASTRIVDGVSTADVL 239
A-HbmAI 160 VMLYTSGTTGLPKGVLSTQRNCLWSLAACYVPVTGLTAEDRVLWPLPLFHSLSHIVCLLAATAVGAGTRIVDGVSTSDVL 239
A-AsmA 152 VMLYTSGTTGAPKGVLSTQRNCLWSVAACYAPVLGLTPDDRVLWPLPLFHSLSHIAGVLATTAVGATTRVVDGRSPQDVL 231
A-RapA 157 VMLYTSGTTGTPKGVLSTQGSGLWSAAYCDIPAWELTENDVLLWPAPLFHRLALHLCLLATTAVGATARIMNGFVASEVL 236
Consensus
WM+YTSGTTGRPKGVLSTQRNCLWSVAACYVP+PGLTADDRVLWPLPLFHSLSHIACVLA+TAVGATARI+DGSSADDVL
A-CxmA 236 DALRAHAPTFLAGVPTTYHHLVTAARQDGFSAPGLRIGLVGGAVTTAALRRDVEETFGVPLVDAYGSTETCGAITMNPPD 315
A-RifA 235 RLIEAESSTFLAGVPTTYHHLVRAARQRGFSAPSLRIGLAGGAVLGAGLRSEFEETFGVPLIDAYGSTETCGAITMNPPD 314
A-NatA 241 DLLRTERATFLAGVPTTYHHLVTTARRTGLSLPDLRIGLVGGAVTGSELRRSFEETFGVPLVDAYGSTETCGAITMNPPD 320
A-RubA 240 EAARETRATFVAGVPTTYHYLLEARRQRRITLPDLRIGLVGGAVAGPGLCRSFREEFGVPLVDAYGSTETCGAITMNPPG 319
A-GdmAI 240 DALREERSTFIAGVPTLYHHLIEAARERDFATPELRIALVGGAVATADLVRSFESAFGVPLVDAYGSTETCGAIAVNW-- 317
A-HbmAI 240 DALREERSTFIAGVPTLYHHLIEAARERDFATPELRIALVGGAVATADLVRSFEATFGVPLVDAYGSTETCGAIAVNWPT 319
A-AsmA 232 AALHEERSTVLAGVPTTYHHLVRAAGERGFLAPDLRVGLVGGAITTAELRAAVEQRFGVPLIDAYGSTETCGSITVNWPT 311
A-RapA 237 EELTEHRCTVLVGVPTMSRYLLGAADTFEPRTSSLKMGLVAGSVAPASLIEGFEDVFGVPLLDTYGCTETSGSLTVNWLS 316
Consensus
DALREERSTFLAGVPTTYHHLVEAAR+RGFS+PDLRIGLVGGAVATA+LRRSFEETFGVPLVDAYGSTETCGAIT+N+PD
TGD
A-CxmA 316 GLRVDGSCGLPVPGVGVRIVDPDTGRDLPAGAEGEVWVSGPNVMVGYHNSPEATAEALRDGWYRTGDLARRDDAGYFTIC 395
A-RifA 315 GARVEGSCGLAVPGVDVRVVDPDTGLDVPAGEEGEVWVSGPNVMLGYHNSPEATAAAMRDGWFRTGDLARRDDAGYFTIC 394
A-NatA 321 GARVDGSCGLPVPGVDVRIVDPDTGDDAPAGKEGEVWVSGPNVMVGYHNSPEATRAALRDGWFRTGDLARRDEAGYFTVC 400
A-RubA 320 GVRVDGSCGLPVPGVDVRIVDPETGRDVPAGAEGEVWVRGPNVTPGYHNKPEATAAAFQDGWYRTGDLARRDAAGYFTIS 399
A-GdmAI 318 ---VT 319
A-HbmAI 320 GPRVEGSCGLPVPGLTVRLVDPDTGVDVPAGREGEFWVSGPNIMAGYHNQPEATAAALRDGWYRTGDLGRRDEAGFCTVT 399
A-AsmA 312 GPRVEGSSGLPVPGLAVRLVDPDTGLDAPTGAEGEVWVRGPSVMVGYHNRPEATAAALRDGWYRTGDLATRDESGFHAVT 391
A-RapA 317 GQRIPGSCGLPVPGLSLRFVDPISGADVADGEEGELWASGPSIMIGYHEQPEATAEVLSDGWYRTGDLARRSETGHVTIT 396
Consensus
G+RV+GSCGLPVPGVDVRIVDPDTG+DVPAGAEGEVWVSGPNVMVGYHNSPEATAAALRDGWYRTGDLARRDEAGYFT+T
A-CxmA 396 GRIKELVIRGGENIHPQEVETVLRTVPGVADAAVAGVPHDTLGEVPVAYVIPGPSGFDVRDLLARCRERLAAYKVPEQIH 475
A-RifA 395 GRIKELIIRGGANIHPGEVEAVLRTVDGVADAAVGGVPHDTLGEVPVAYVIPGPTGFDPAALIEKCREQLSAYKVPDRIL 474
A-NatA 401 GRIKDLVVRGGENIHPDEVEAVLRTAPGVADAGVAGAAHDTLGEVPVAWLVPGPDGLDIAAVLDHCRARLSAYKIPERIH 480
A-RubA 400 GRINDLIVRGGENVHPEEIEAVIRAVPGIADVGVAGRPHEVLGEVPVAYVVAGPSGVEADAVIERCRRELSTFKLPEEVY 479
A-GdmAI 320 GRIKELVIRAGENIHPGEVEAVLRTVPGVADAAVVGKPHAVLGEVPVAFVVPGPDGFDPSALLATCRERLSYFKVPEEIY 399
A-HbmAI 400 GRIKELVIRAGENIHPGEVEAVLRTVPGVADAAVVGKPHAVLGEVPVAFVVPGPDGFDPSALLATCRERLSYFKVPEEIY 479
A-AsmA 392 GRIDDVVVRGGEKVHPAEVEAVLRAVPGVADAAVVGRPHDVLGEVPVAFLVPG-EGFDPAQVLAVCRERLSYHKVPEELY 470
A-RapA 397 GRIKELIIRGGENIHPHEIEAVALDVPGVKDAAAAGKQHPVLGEIPVLYVVPETGGVDTDMVLAVCRERLSYFKVPEEIY 476
Consensus
GRIKELVIRGGENIHPGEVEAVLRTVPGVADAAVAGKPHDVLGEVPVAYVVPGPDGFDPAA+LA+CRERLSYFKVPEEIY
A-CxmA 476 EVAAIPRTASGKTRRRLL 493
A-RifA 475 EVAHIPRTASGKIRRGL- 491
A-NatA 481 EAAGIPRTPSGKIVRRLL 498
A-RubA 480 EVAGVPRTASGKIQRRLL 497
A-GdmAI 400 EIARVPRTASGKITRHVL 417
A-HbmAI 480 EIARVPRTASGKITRHVL 497
A-AsmA 471 QIESVPRTASGKITRRVL 488
A-RapA 477 RVDAIPRTASGKVKRSSL 494
Consensus
EVA++PRTASGKITRRLL

Adenylation domain	Biosynthetic gene cluster	Substrate specificity	Microorganism
A-CxmA	Chaxamycin	AHBA	*Streptomyces leeuwenhoekii*
A-RifA	Rifamycin	AHBA	*Amycolatopsis mediterranei* S699
A-NatA	Naphthomycin	AHBA	*Streptomyces* sp. CS
A-RubA	Rubradirin	AHBA	*Streptomyces achromogenes* var. rubradiris NRRL 3061
A-GdmAI	Geldanamycin	AHBA	*Streptomyces geldanamycininus* NRRL 3602
A-HbmAI	Herbimycin	AHBA	*Streptomyces hygroscopicus* AM 3672
A-AsmA	Ansamitocin	AHBA	*Actinosynnema pretiosum* subsp. auranticum ATCC 31565
A-RapA	Rapamycin	DHCHC	*Streptomyces rapamycinicus* NRRL 5491

AHBA = 3-amino-5-hydroxybenzoic acid; DHCHC = 4,5-dihydroxycyclohex-1-ene carboxylic acid.

FIG S9 Comparison of the amino acid sequence of the adenylation domain present in the loading module of the chaxamycin polyketide synthase (PKS) with other adenylation domains from PKSs of known specificity. The amino acid sequences were identified by Conserved Domains Search at NCBI (8) and aligned in ClustalX (9) and visualised in Jalview (10). The AMP-binding [LIVMFY]-{E}-{VES}-[STG]-[STAG]-G-[ST]-[STEI]-[SG]-x-[PASLIVM]-[KR] motif (Prosite (11) accession number PS00455) and the conserved ATPase TGD motif (12) are shown in yellow boxes.

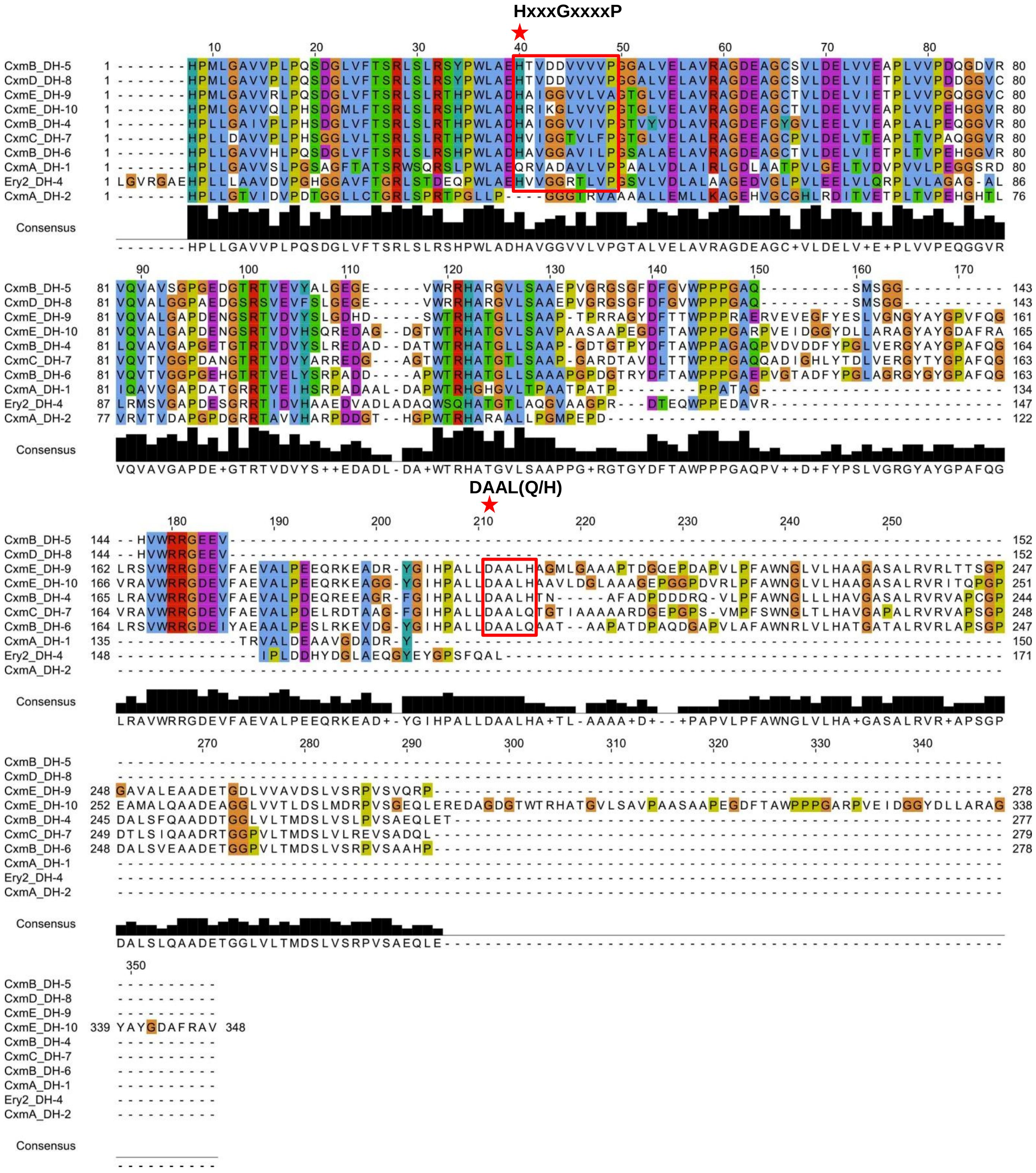
HxxxGxxxxP
DAAL(Q/H)
CxmB_DH-5
CxmD_DH-8
CxmE_DH-9
CxmE_DH-10
CxmB_DH-4
CxmC_DH-7
CxmB_DH-6
CxmA_DH-1
Ery2_DH-4
CxmA_DH-2
Consensus

FIG S10 Manually-curated bioinformatic analysis of DH domains present in the chaxamycin PKS to assess dehydratase activity. The amino-acid sequences of DH domains detected by Conserved Domains Search at NCBI (8) were aligned in ClustalX (9) and visualised in Jalview (10). The conserved catalytic dyad present in DH-4 from the erythromycin polyketide synthase consists of the His residue in **H**XXXGXXXXP motif and the Asp in **D**(A/V)(V/A)(A/L)(Q/H) (13). The conserved motifs are shown in red boxes and the His and Asp residues of the catalytic dyad are indicated with red stars.

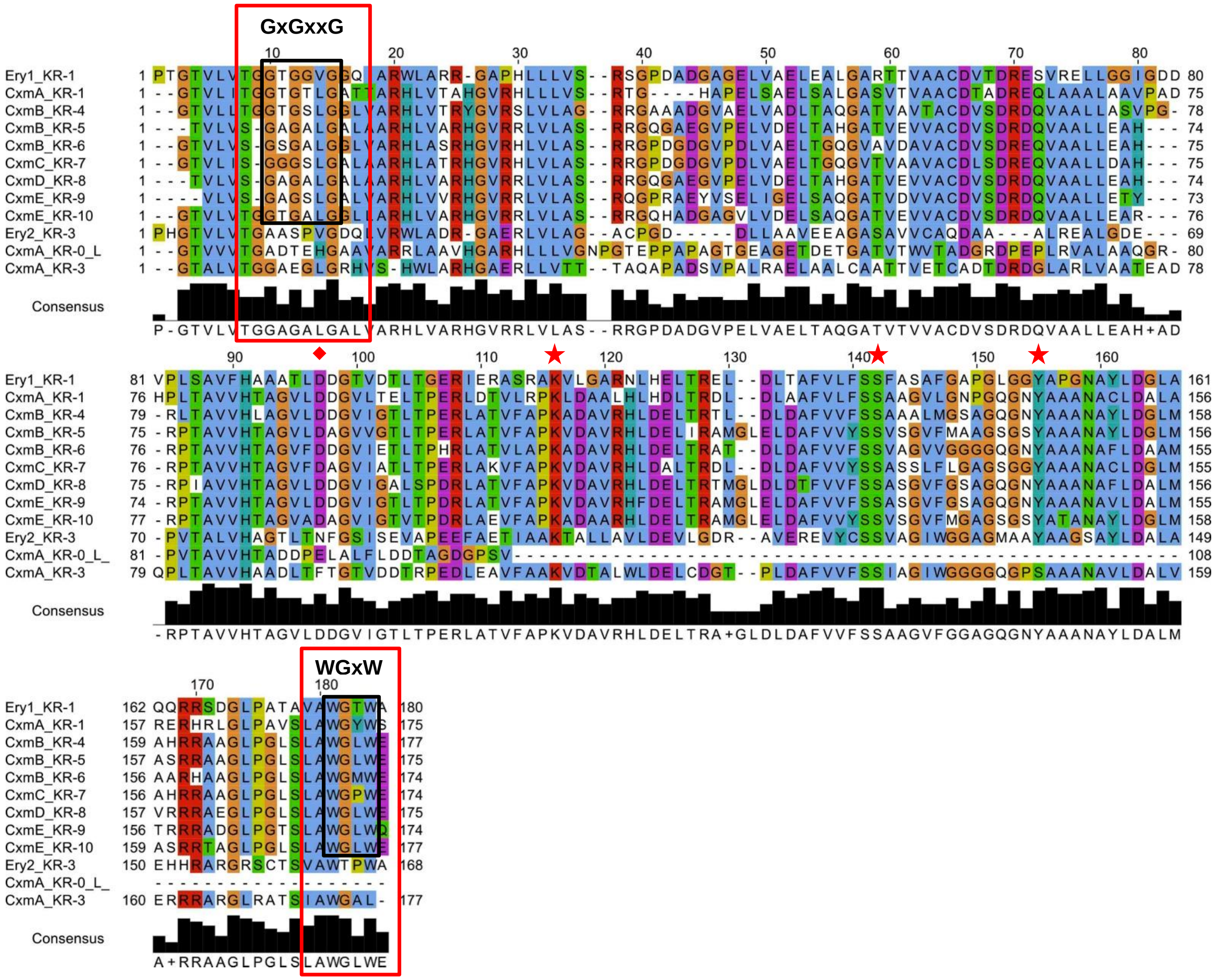

FIG S11 Manually-curated bioinformatic analysis of KR domains present in the chaxamycin PKS to assess reductase activity. The amino-acid sequences of KR domains detected by antiSMASH (14) were aligned in ClustalX (9) and visualised in Jalview (10). The NADPH binding motifs previously characterised are shown contained in red boxes; the conserved residues in the KR domains with active reductase activity, GxGxxG and WGxW, are contained in black boxes (as shown in Figure 2 of Reference (15)). Erythromycin KR domains 1 and 3 are shown for reference, as in Figure 2 of Reference (15). A red diamond indicates the Asp residue diagnostic of PKS KR stereospecificity (present = reduction leaves the β-OH group in *D*-orientation; absent = reduction leaves the β-OH group in *L*-orientation) and the catalytic triad in the active site, Lys, Ser and Tyr, are indicated with red stars (as in Reference (16)).

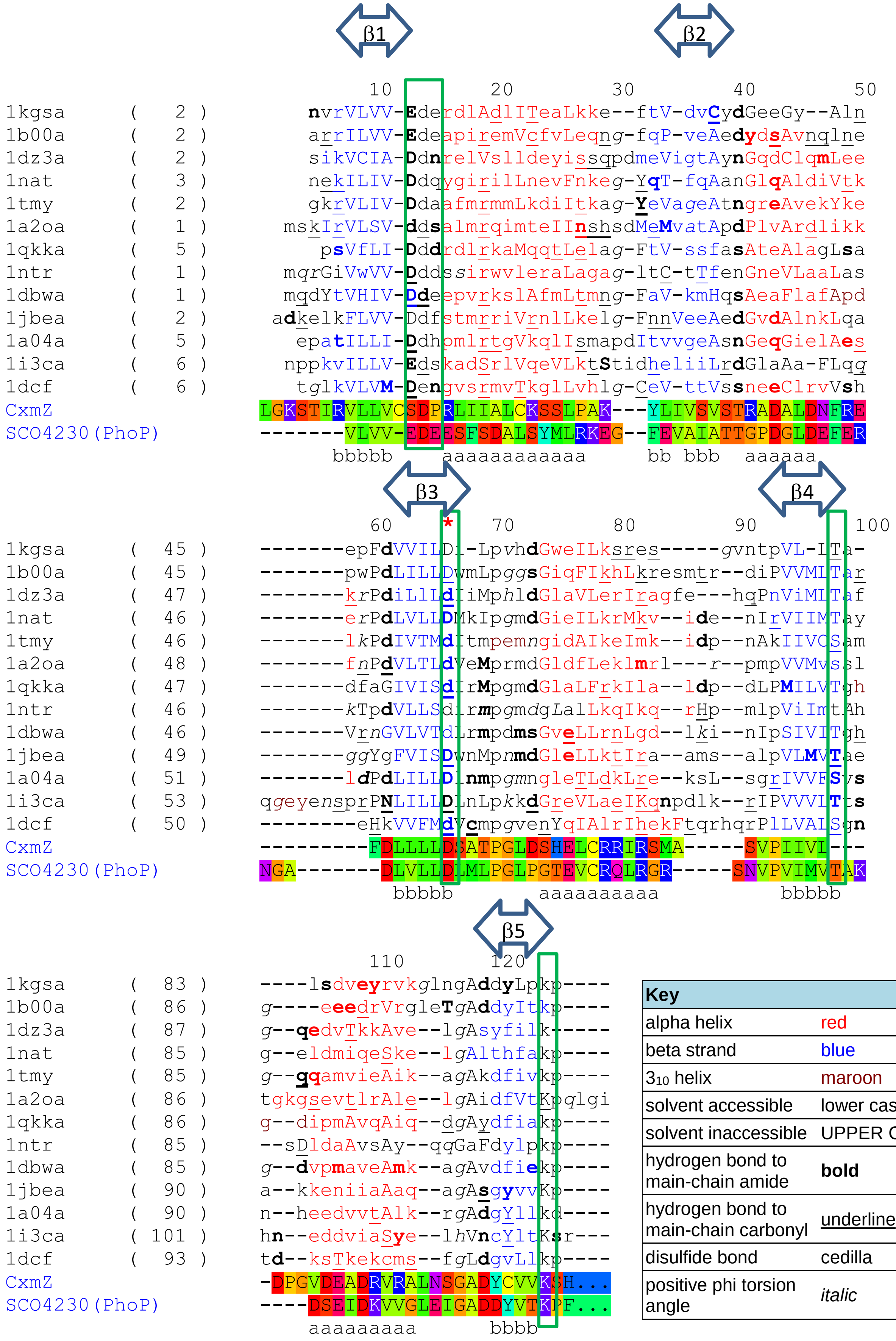

Key		
alpha helix	red	x
beta strand	blue	x
3_{10} helix	maroon	x
solvent accessible	lower case	x
solvent inaccessible	UPPER CASE	X
hydrogen bond to main-chain amide	**bold**	**x**
hydrogen bond to main-chain carbonyl	underline	x
disulfide bond	cedilla	ç
positive phi torsion angle	*italic*	*x*

FIG S12 Predicted secondary structure of CxmZ and evaluation of the presence of catalytically-relevant residues, supporting the classification of CxmZ as an Atypical Response Regulator. Structural alignment of CxmZ, with several response regulators whose tertiary structure has been solved, performed with FUGUE (Ver 2.0) Profile Library Search Against HOMSTRAD (http://tardis.nibio.go.jp/fugue/align.php; (17). The five conserved catalytic residues (18) at the carboxy-termini of the beta-sheets (Ct-β) are boxed in green: three acidic residues at the end of Ct-β1 (D,D,D/E), the aspartate that is phosphorylated at the end of Ct-β3 (red asterisk), the threonine or serine at the end of Ct-β4 and the lysine at the end of Ct-β5. The typical-response-regulator PhoP from *Streptomyces coelicolor* (CAB77324.1) has been aligned for reference. Note that two of the acidic residues at the end of Ct-β1 involved in Mg^{2+} binding are missing in CxmZ, making phosphorylation of the Asp residue at Ct-β3 unlikely. The conserved catalytic site threonine/serine at Ct-β4 is also missing in CxmZ.

PDB accession number	Function	Microorganism
1kgs	DNA binding response regulator D	*Thermotoga maritima*
1b00	Phosphate regulon transcriptional regulatory protein PhoB	*Escherichia coli*
1dz3	Stage 0 sporulation protein A	*Bacillus stearothermophilus*
1nat	Sporulation response regulatory protein	*Bacillus subtilis*
1tmy	CheY protein	*Thermotoga maritima*
1a2o	CheB methylesterase	*Salmonella typhimurium*
1qkk	C4-dicarboxylate transport transcriptional regulatory protein, receiver domain	*Sinorhizobium meliloti*
1ntr	N-terminal receiver domain of NtrC	*Salmonella typhimurium*
1dbw	Transcriptional regulatory protein FixJ, receiver domain	*Rhizobium meliloti*
1jbe	Chemotaxis protein CheY	*Escherichia coli*
1a04	Nitrate/nitrite response regulator protein NarL, N-terminal domain	*Escherichia coli*
1i3c	Response regulator Rcp1	*Synechosystis* Sp. Pcc 6803
1dcf	Etr1 protein, receiver domain	*Arabidopsis thaliana*

```
Sle29840     412 SIKEFFGTSQLSQFMDQNNPLSGLTHKRRLNALGPGGLSRERAGFEVRDVHPSHYGRMCP 471
SCO4654      412 SIKEFFGTSQLSQFMDQNNPLSGLTHKRRLNALGPGGLSRERAGFEVRDVHPSHYGRMCP 471
Ame_rpoB     417 AIKEFFGTSQLSQFMQQTNPIDGLTHKRRLNALGPGGLSRERAGMEVRDVHPSHYGRMCP 476
                 :**************:*.**:.**********************:***************
```

FIG S13 Alignment of RpoB sequences (with ClustalX; (9)) showing the region that contains the amino-acid residues responsible for resistance to rifamycin in the natural producer *Amycolatopsys mediterranei* S699: Q432, D438 and N447 (19) are highlighted in green. Sle29840, *Streptomyces leeuwenhoekii*; SCO4654, *Streptomyces coelicolor* A3(2) (NP_628815.1), Ame_rpoB, *A. mediterranei* S699 (AAS07760.1). The alignment reveals that the residues at equivalent positions 432 and 438 in *S. leeuwenhoekii* are the same as those in the RpoB of *S. coelicolor*, which is rifamycin-sensitive.

TABLE S1 DNA sequences of phage integration sites.

attB site	DNA sequence 5'➔3'
ΦC31-Sco	CGGTGCGGGTGCCAGGGCGTGCCC TT GGGCTCCCCGGGCGCGTACTCCAC
ΦC31-Sle	CGGTGGGGGTGCCAGGGGGTGCCC TT GGGCTCTCCGGGCGCGTACTCCAC
ΦBT1-Sco	TCCTTGACCAGGTTTTTGACGAAA GT GATCCAGATGATCCAGCTCCACAC
ΦBT1-Sle	TCCTTGATCAGATTTTTGACGAAA GT GATCCAGATGACCCAGCTCCACAC

Putative DNA sequences of bacterial attachment sites (*attB*) that are recognised by the ΦC31 and ΦBT1 integrases in *S. leeuwenhoekii* (Sle) were identified using BLASTn (20) with the known *attB* sequences for ΦC31 and ΦBT1 in *S. coelicolor* (Sco) (21, 22), respectively). Differences are underlined.

TABLE S2 Genes in which phage integration sites are located

attB site	Locus coordinates	Gene	Predicted gene product function (annotation)
ΦC31-Sco	260747..260796	*sco3798*	Pirin protein (chromosome condensation protein)
ΦC31-Sle	4154940..4154989	*sle35040*	Pirin protein (putative quercetin 2,3-dioxygenase PA2418)
ΦBT1-Sco	201997..201948	*sco4848*	Putative integral membrane protein
ΦBT1-Sle	3403179..3403228	*sle28260*	Putative integral membrane protein

The genes in which the ΦC31 and ΦBT1 attachment sites (*attB*) are located in the *S. coelicolor* (Sco) and *S. leeuwenhoekii* (Sle) chromosomes are homologs. Sle35040 and Sle28260 have 92% and 91% amino acid identity with Sco3798 (Gene ID: 1099234) and Sco4848 (Gene ID: 1100289), respectively.

TABLE S3: Identity between proteins encoded by the chaxamycin (*cxm*) and other ansamycin-type biosynthetic gene clusters.

Protein	Protein length	Highest identity[1]	Rifamycin (*rif*)[2]	Saliniketal/ Rifamycin (*sare*)[3]	Naphthomycin (*nat*)[4]	Rubradirin (*rub*)[5]	Ansamitocin (*asm*)[6]	Geldanamycin (*gdm*)[7]
Sle10440	693	Alpha-galactosidase (melibiase), highly conserved but no *Streptomyces coelicolor* homolog; *Streptomyces cellulosae* (WP_030676983.1); 599/694 (86)						
Sle10430	326	Peptidase, highly conserved, 71% identity; ***Streptomyces coelicolor* Sco6773**						
Sle10420	261	GDSL-like lipase/acylhydrolase, very highly conserved, 92% identity; ***Streptomyces coelicolor* Sco6774**						
Sle10410	201	Carboxymuconolactone decarboxylase, highly conserved but no *Streptomyces coelicolor* homolog; *Streptomyces*						

Protein	Protein length	Highest identity[1]	Rifamycin (*rif*)[2]	Saliniketal/ Rifamycin (*sare*)[3]	Naphthomycin (*nat*)[4]	Rubradirin (*rub*)[5]	Ansamitocin (*asm*)[6]	Geldanamycin (*gdm*)[7]
		sp. NRRL F-4835 (WP_030971727.1); 167/191 (87)						
Sle10400	398	Metallo-beta-lactamase, very highly conserved, 84% identity; ***Streptomyces coelicolor* Sco6776**						
Cxm1	138	Hypothetical protein; *Streptomyces turgidiscabies* (WP_006375853.1); 55/71 (77)						
CxmS	327	Dehydrogenase; *Amycolatopsis mediterranei* (KDO09658.1); 260/327 (80)	RifS - Putative NADH-dependent dehydrogenase (AAS07752.1); 255/322 (79)	Sare1242 - Oxidoreductase domain protein (ABV97147.1); 246/325 (76)				Orf6 - Oxidoreductase-like (AAY46807.1); 36/132 (27)
CxmT	323	Oxidoreductase; *Streptomyces katrae* (WP_030301473.1); 204/326 (63)	RifT - NADH-dependent dehydrogenase (AAC01707.1); 141/265 (53)	Sare1243 - Oxidoreductase domain protein (ABV97148.1); 190/322 (59)				
Cxm4	397	Cytochrome P450; *Actinomadura rifamycini* (WP_026404209.1); 324/397 (82)	Orf0 - Cytochrome P450-like protein (AAC01709.1); 320/397 (81)	Sare1245 - Cytochrome P450 (ABV97150.1); 320/397 (81)	Orf0 - Cytochrome P450-like protein (ADM46355.1); 269/397 (68)	RubF4 - Putative cytochrome P450 (CAI94704.1); 190/408 (47)	Asm30 - Cytochrome P450 (AAM54108.1); 63/202 (31)	GdmP - P450 (PikC subfamily) (AAO06929.1); 149/391 (38)
CxmA	5616	AMP-dependent	RifA - Rifamycin polyketide	Sare1246 - AMP-dependent	NatA - Polyketide synthase	RubA - Putative polyketide	AsmD - Polyketide Synthase	GdmAII - Polyketide

Protein	Protein length	Highest identity[1]	Rifamycin (*rif*)[2]	Saliniketal/ Rifamycin (*sare*)[3]	Naphthomycin (*nat*)[4]	Rubradirin (*rub*)[5]	Ansamitocin (*asm*)[6]	Geldanamycin (*gdm*)[7]
		synthetase and ligase; *Salinispora arenicola* (WP_012181459.1); 3525/4669 (75)	synthase (AAC01710.1); 1936/2678 (72)	synthetase and ligase (ABV97151.1); 3525/4669 (75)	(ADM46356.1); 3425/5078 (67)	synthase (CAI94682.1); 2207/3714 (59)	(AAM54078.1); 1488/2991 (50)	synthase; modules 4-5 (AAO06917.1); 1395/2703 (52)
CxmB	5363	Rifamycin polyketide synthase; *Salinispora arenicola* (WP_019902108.1); 3969/5273 (75)	RifB - Rifamycin polyketide synthase (AAC01711.1); 3854/5166 (75)	Sare1247 - Beta-ketoacyl synthase (ABV97152.1); 3969/5273 (75)	NatC – Polyketide synthase (ADM46358.1); 3560/5315 (67)	RubB - Putative polyketide synthase (CAI94713.1); 2550/4688 (54)	AsmD - Polyketide synthase (AAM54078.1); 1208/2218 (54)	GdmAII - Polyketide synthase; modules 4-5 (AAO06917.1); 1485/2730 (54)
CxmC	1820	Rifamycin polyketide synthase; *Amycolatopsis mediterranei* (WP_013222549.1); 1361/1827 (74)	RifC - Rifamycin polyketide synthase (AAC01712.2); 1365/1823 (75)	Sare1248 - Beta-ketoacyl synthase (ABV97153.1); 1329/1844 (72)	NatC – Polyketide synthase (ADM46358.1); 1126/1779 (63)	RubB - Putative polyketide synthase (CAI94713.1); 993/1787 (56)	AsmD - Polyketide synthase (AAM54078.1); 910/1749 (52)	GdmAII - Polyketide synthase; modules 4-5 (AAO06917.1); 950/1823 (52)
CxmD	1773	Polyketide synthase; *Streptomyces albogriseolus* (AHD24377.1); 1313/1864 (70)	RifD - Rifamycin polyketide synthase (AAC01713.1); 1281/1788 (72)	Sare1249 - Beta-ketoacyl synthase (ABV97154.1); 1252/1817 (69)	NatC – Polyketide synthase (ADM46358.1); 1082/1778 (61)	RubB - Putative polyketide synthase (CAI94713.1); 967/1777 (54)	AsmD - Polyketide Synthase (AAM54078.1); 899/1731 (52)	GdmAII - Polyketide synthase; modules 4-5 (AAO06917.1); 936/1808 (52)
CxmE	3488	Polyketide synthase; *Salinispora arenicola* (WP_028678212.1); 2468/3522 (70)	RifE - Rifamycin polyketide synthase (AAC01714.1); 2434/3497 (70)	Sare1250 - Acyl transferase (ABV97155.1); 2463/3521 (70)	NatC – polyketide synthase (ADM46358.1); 2189/3571 (61)	RubB - Putative polyketide synthase (CAI94713.1); 1825/3557 (51)	AsmD - Polyketide synthase (AAM54078.1); 1154/2199 (52)	GdmAII - Polyketide synthase; modules 4-5 (AAO06917.1); 1357/2757 (49)
CxmF	269	*N*-acetyltransferase/amide synthase; *Amycolatopsis*	RifF - Amide synthase (AAC01715.1); 194/256 (76)	Sare1251 - *N*-acetyltransferase (ABV97156.1); 185/257 (72)	NatF - Amide synthase (ADM46361.1); 158/289 (55)	RubF - Putative amide synthase (CAI94702.1); 125/267 (47)	Asm9 - Amide synthase (AAM54087.1); 105/258 (41)	GdmF - amide synthase; RifF homolog (AAO06919.1); 107/273 (39)

Protein	Protein length	Highest identity[1]	Rifamycin (*rif*)[2]	Saliniketal/ Rifamycin (*sare*)[3]	Naphthomycin (*nat*)[4]	Rubradirin (*rub*)[5]	Ansamitocin (*asm*)[6]	Geldanamycin (*gdm*)[7]
		vancoresmycina (WP_004559807.1); 189/257 (74)						
CxmG	368	Aminodehydroquinate synthase; *Amycolatopsis mediterranei* U32 (YP_003762846.1); 293/347 (84)	RifG - Aminodehydroquinate synthase (AAC01717.1); 290/341 (85)	Sare1253 - 3-dehydroquinate synthase (ABV97158.1); 284/341 (83)	NatG - 3-dehydroquinate synthase (ADM46363.1); 266/336 (79)	RubG - Putative aDHQ synthase (CAI94724.1); 266/332 (80)	Orf10 - Aminodehydroquinate synthase (AAC14006.1); 246/335 (73)	Orf2 - Aminodehydroquinate synthase (AAY46803.1); 243/332 (73)
CxmH	406	Phospho-2-dehydro-3-deoxyheptonate aldolase; *Amycolatopsis vancoresmycina* (WP_004559804.1); 311/413 (75)	RifH - AminoDAHP synthase (AAC01718.1); 290/427 (68)	Sare1254 - 3-deoxy-7-phosphoheptulonate synthase (ABV97159.1); 291/391 (74)	NatH - DAHP synthase (ADM46364.1); 272/441 (62)	RubH - Putative DAHP synthase (CAI94725.1); 295/397 (74)		
CxmI	268	Shikimate dehydrogenase; *Actinomadura rifamycini* (WP_026404009.1); 213/266 (80)	RifI - Aminoquinate dehydrogenase (AAC01719.1); 202/261 (77)	Sare1274 - Shikimate dehydrogenase substrate binding domain protein (ABV97179.1); 144/265 (54)	NatI - Shikimate/quinate dehydrogenase (ADM46365.1); 186/268 (69)	RubI - Putative oxidoreductase (CAI94690.1); 18/44 (41)		
CxmK	386	3-amino-5-hydroxybenzoic acid synthase; *Amycolatopsis mediterranei*	RifK - AHBA synthase (AAC01720.1); 330/388 (85)	Sare1255 - Glutamine--scyllo-inositol transaminase (ABV97160.1); 321/388 (83)	NatK - 3-amino-5-hydroxybenzoic acid synthase (ADM46366.1); 313/384 (82)	RubK - Putative AHBA synthase (CAI94689.1); 293/384 (76)	Orf6 - 3-amino-5-hydroxybenzoic acid synthase (AAC13997.1); 270/365 (74)	Orf5 – AHBA synthase (AAY46806.1); 281/385 (73)

Protein	Protein length	Highest identity[1]	Rifamycin (*rif*)[2]	Saliniketal/ Rifamycin (*sare*)[3]	Naphthomycin (*nat*)[4]	Rubradirin (*rub*)[5]	Ansamitocin (*asm*)[6]	Geldanamycin (*gdm*)[7]
		(AAA75105.1); 329/388 (85)						
CxmL	358	Oxidoreductase; *Streptomyces* sp. 192 (ADI56541.1); 269/356 (76)	RifL - Putative oxidoreductase (AAS07754.1); 268/357 (75)	Sare1256 - Oxidoreductase domain protein (ABV97161.1); 265/357 (74)	NatL – Oxidoreductase (ADM46367.1); 232/359 (65)	RubL - Putative oxidoreductase (CAI94690.1); 199/345 (58)	Orf7 – Oxidoreductase (AAC14003.1); 203/365 (56)	Orf6 - Oxidoreductase-like (AAY46807.1); 207/362 (57)
CxmM	232	Phosphoglycolate phosphatase; *Amycolatopsis rifamycinica* (KDN20697.1); 191/232 (82)	RifM - Putative phosphatase (AAC01721.1); 193/232 (83)	Sare1257 - AHBA synthesis associated protein (ABV97162.1); 185/232 (80)	NatM – Phosphatase (ADM46368.1); 187/231 (81)	RubM - Putative phosphatase (CAI94691.1); 146/215 (68)	Orf8 – Phosphatase (AAC14004.1); 140/204 (69)	Ahba1b - Phosphatase-like (AAY46808.1); 151/213 (71)
CxmN	307	Kanamycin kinase; *Streptomyces katrae* (WP_030293822.1); 225/295 (76)	RifN - Kanosamine kinase (AAC01722.2); 164/230 (71)	Sare1275 - ROK family protein (RifN AHBA kinase) (ABV97180.1); 157/290 (54)	NatN - Glucose kinase (ADM46369.1); 194/292 (66)	RubN - Putative kinase (CAI94692.1); 159/298 (53)	Asm22 – Kinase (AAM54100.1); 122/226 (54)	Orf3 - Kinase-like (AAY46804.1); 150/253 (59)
Cxm18	295	N5,N10-methylene tetrahydromethanopterin reductase; *Amycolatopsis mediterranei* (WP_013222573.1); 204/295 (69)	Orf11 - Putative flavin-dependent oxidoreductase (AAC01735.3); 204/295 (69)			Orf1 – Hypothetical protein (CAI94678.1); 88/269 (33)		
Cxm19	533	3-(3-hydroxyphenyl) propionate hydroxylase; *Streptomyces*	Orf19 - Putative 3-(3-hydroxylphenyl) propionate hydroxylase (AAG52989.1);	Sare1268 - Monooxygenase FAD-binding (ABV97173.1); 405/518 (78)	Nat2 - FAD-dependent oxidoreductase (ADM46370.1); 367/540 (68)	RubP1 - Putative hydroxyphenylpropionate (CAI94716.1); 305/522 (58)	Asm11 – Oxygenase (AAM54089.1); 86/273 (32)	GdmM - Similar to Rif19; putative FAD-dependent monooxygenase (AAO06920.1);

Protein	Protein length	Highest identity[1]	Rifamycin (*rif*)[2]	Saliniketal/ Rifamycin (*sare*)[3]	Naphthomycin (*nat*)[4]	Rubradirin (*rub*)[5]	Ansamitocin (*asm*)[6]	Geldanamycin (*gdm*)[7]
		katrae (WP_030293823.1); 429/526 (82)	388/519 (75)					279/531 (53)
Cxm20	402	Acyltransferase; *Salinispora arenicola* (WP_029537705.1); 253/407 (62)	Orf20 - Putative polyketide associated protein (AAG52990.1); 252/404 (62)	Sare1262 - Conserved polyketide synthase associated protein PapA5 (ABV97167.1); 249/407 (61)				
Cxm21	63	Ferredoxin; *Streptomyces chartreusis* (WP_010040569.1); 43/63 (68)		Sare1261 – Protein of unknown function DUF1271 (SCO7676 ferredoxin) (ABV97166.1); 22/64 (34)				
Cxm22	393	Cytochrome P450; *Streptomyces* sp. NRRL S-118 (WP_031068103.1); 258/399 (65)	Orf4 - Putative cytochrome P450 oxidoreductase (AAC01728.1); 221/403 (55)	Sare1260 - Cytochrome P450 (ABV97165.1); 154/394 (39)	Orf0 - Cytochrome P450-like protein (ADM46355.1); 143/404 (35)	RubF4 - Putative cytochrome P450 (CAI94704.1); 121/410 (30)	Asm30 - Cytochrome P450 (AAM54085.1); 45/186 (24)	GdmP - P450 (PikC subfamily) (AAO06929.1); 177/409 (43)
Cxm23	418	Cytochrome P450; *Salinispora arenicola* (WP_029021601.1); 322/412 (78)	Orf13 - Putative cytochrome P450 monooxygenase (AAC01737.2); 306/423 (72)	Sare1259 - Cytochrome P450 (ABV97164.1); 322/412 (78)	Orf0 - Cytochrome P450-like protein (ADM46355.1); 135/405 (33)	RubP6 - Cytochrome P450 (CAI94681.1); 144/372 (39)	Asm30 - Cytochrome P450 (AAM54085.1); 93/327 (28)	GdmP - P450 (PikC subfamily) (AAO06929.1); 135/404 (33)
Cxm24	355	Methyltransferase; *Streptomyces* sp. CNH189	Orf14 - C-27 *O*-methyltransferase (AAC01738.1); 22/87 (25)				Asm7 – Methyltransferase (AAM54085.1); 53/224 (24)	

Protein	Protein length	Highest identity[1]	Rifamycin (*rif*)[2]	Saliniketal/ Rifamycin (*sare*)[3]	Naphthomycin (*nat*)[4]	Rubradirin (*rub*)[5]	Ansamitocin (*asm*)[6]	Geldanamycin (*gdm*)[7]
		(AGH68906.1); 230/356 (65)						
CxmY	433	Transcriptional regulator; *Streptomyces albogriseolus* (AHD24357.1); 289/430 (67)	Orf36 - Putative regulatory protein (AAS07758.1); 182/416 (44)	Sare1270 - Transcriptional regulator, LuxR family (ABV97175.1); 177/416 (43)	Orf5 - Transcriptional regulator (ADM46353.1); 184/384 (48)	RubRg2 - Putative transcription regulator (CAI94711.1); 113/332 (34)		
CxmZ	246	Hypothetical protein - Response regulators OmpR; *Streptomyces* sp. NRRL B-1347 (WP_030679122.1); 177/242 (73)						
CxmJ	168	3-dehydroquinate dehydratase; *Streptomyces katrae* (WP_030293833.1); 130/147 (88)	RifJ - Aminodehydroquinate dehydratase (AAS07762.1); 133/153 (86)	Sare1277 - 3-dehydroquinate dehydratase (ABV97182.1); 113/140 (81)	NatJ - Aminodehydroquinate dehydratase (ADM46373.1); 104/130 (80)	RubJ - Putative aDHQ dehydratase (CAI94721.1); 108/147 (73)	Asm23 - 5-deoxy-5-amino-3-dehydroquinate dehydratase (AAM54101.1); 94/127 (74)	Orf4 - aDHQ dehydratase (AAY46805.1); 98/144 (68)

In order to assess the similarity of the chaxamycin biosynthetic gene cluster (*cxm*) to those for other ansamycin-type natural products, and to infer possible chaxamycin biosynthetic reactions by comparison with known biosynthetic pathways, we used BLASTp (20) to search for similar proteins to those encoded by the *cxm* cluster, either searching the NCBI non-redundant database or with searches targeted at specific ansamycin-type biosynthetic gene clusters. The information given in each cell of the table is, in this order: annotated function; species (accession number); identical residues/total alignment (%identity).

[1]Protein found in the non-redundant database from NCBI (accessed on 13 August 2014) with highest identity; [2]rifamycin biosynthetic gene cluster (*rif*) from *Amycolatopsis mediterranei* S699 (AF040570.3); [3]saliniketal/rifamycin biosynthetic gene cluster (*sare*) from *Salinispora arenicola* strain CNS-205 (CP000850); [4]naphthomycin biosynthetic gene cluster (*nat*) from *Streptomyces* sp. CS (GQ452266.1); [5]rubradirin biosynthetic gene cluster (*rub*) from *Streptomyces achromogenes* subsp. *rubradiris* (AJ871581.1); [6]ansamitocin biosynthetic gene cluster (*asm*) from *Actinosynnema pretiosum* subsp. *auranticum* (AF453501.1 and U33059.1); [7]geldanamycin biosynthetic gene cluster (*gdm*) from *Streptomyces geldanamycininus* (AY952143 and AY179507).

References

1. **Kieser T**, **Bibb MJ**, **Buttner MJ**, **Chater KF**, **Hopwood DA**. 2000. Practical *Streptomyces* Genetics. John Innes Foundation, Norwich, UK.
2. **Rateb ME**, **Houssen WE**, **Arnold M**, **Abdelrahman MH**, **Deng H**, **Harrison WTA**, **Okoro CK**, **Asenjo JA**, **Andrews BA**, **Ferguson G**, **Bull AT**, **Goodfellow M**, **Ebel R**, **Jaspars M**. 2011. Chaxamycins A–D, bioactive ansamycins from a hyper-arid desert *Streptomyces* sp. J Nat Prod **74**:1491–1499.
3. **Floss HG**, **Yu T-W**, **Arakawa K**. 2011. The biosynthesis of 3-amino-5-hydroxybenzoic acid (AHBA), the precursor of mC_7N units in ansamycin and mitomycin antibiotics: a review. J Antibiot **64**:35–44.
4. **Yuan H**, **Zhao W**, **Zhong Y**, **Wang J**, **Qin Z**, **Ding X**, **Zhao G-P**. 2011. Two genes, *rif15* and *rif16*, of the rifamycin biosynthetic gene cluster in *Amycolatopsis mediterranei* likely encode a transketolase and a P450 monooxygenase, respectively, both essential for the conversion of rifamycin SV into B. Acta Biochim Biophys Sin (Shanghai) **43**:948–956.
5. **Wilson MC**, **Gulder TAM**, **Mahmud T**, **Moore BS**. 2010. Shared biosynthesis of the saliniketals and rifamycins in *Salinispora arenicola* is controlled by the *sare1259*-encoded cytochrome P450. J Am Chem Soc **132**:12757–12765.
6. **Guo J**, **Frost JW**. 2004. Synthesis of aminoshikimic acid. Org Lett **6**:1585–1588.
7. **Yu TW**, **Müller R**, **Muller M**, **Zhang X**, **Draeger G**, **Kim CG**, **Leistner E**, **Floss HG**. 2001. Mutational analysis and reconstituted expression of the biosynthetic genes involved in the formation of 3-amino-5-hydroxybenzoic acid, the starter unit of rifamycin biosynthesis in *Amycolatopsis mediterranei* S699. J Biol Chem **276**:12546–12555.
8. **Marchler-Bauer A**, **Derbyshire MK**, **Gonzales NR**, **Lu S**, **Chitsaz F**, **Geer LY**, **Geer RC**, **He J**, **Gwadz M**, **Hurwitz DI**, **Lanczycki CJ**, **Lu F**, **Marchler GH**, **Song JS**, **Thanki N**, **Wang Z**, **Yamashita RA**, **Zhang D**, **Zheng C**, **Bryant SH**. 2015. CDD: NCBI's conserved domain database. Nucleic Acids Res **43**:D222–D226.
9. **Thompson JD**, **Gibson TJ**, **Plewniak F**, **Jeanmougin F**, **Higgins DG**. 1997. The CLUSTAL_X windows interface: flexible strategies for multiple sequence alignment aided by quality analysis tools. Nucleic Acids Res **25**:4876–4882.
10. **Waterhouse AM**, **Procter JB**, **Martin DMA**, **Clamp M**, **Barton GJ**. 2009. Jalview Version 2—a multiple sequence alignment editor and analysis workbench. Bioinformatics **25**:1189–1191.
11. **Sigrist CJA**, **de Castro E**, **Cerutti L**, **Cuche BA**, **Hulo N**, **Bridge A**, **Bougueleret L**, **Xenarios I**. 2012. New and continuing developments at PROSITE. Nucleic Acids Res **41**:D344–D347.
12. **Huang G**, **Zhang L**, **Birch RG**. 2001. A multifunctional polyketide–peptide synthetase essential for albicidin biosynthesis in *Xanthomonas albilineans*. Microbiology **147**:631–642.
13. **Valenzano CR**, **You Y-O**, **Garg A**, **Keatinge-Clay A**, **Khosla C**, **Cane DE**. 2010. Stereospecificity of the dehydratase domain of the erythromycin polyketide synthase. J Am Chem Soc **132**:14697–14699.
14. **Blin K**, **Medema MH**, **Kazempour D**, **Fischbach MA**, **Breitling R**, **Takano E**, **Weber T**. 2013. antiSMASH 2.0—a versatile platform for genome mining of secondary metabolite producers. Nucleic Acids Res **41**:W204–W212.
15. **Zheng J**, **Keatinge-Clay AT**. 2011. Structural and functional analysis of C2-type ketoreductases from modular polyketide synthases. J Mol Biol **410**:105–117.
16. **Reid R**, **Piagentini M**, **Rodriguez E**, **Ashley G**, **Viswanathan N**, **Carney J**, **Santi DV**, **Hutchinson CR**, **McDaniel R**. 2003. A model of structure and catalysis for ketoreductase domains in modular polyketide synthases. Biochemistry **42**:72–79.
17. **Shi J**, **Blundell TL**, **Mizuguchi K**. 2001. FUGUE: sequence-structure homology recognition using environment-specific substitution tables and structure-dependent gap penalties. J Mol Biol **310**:243–257.
18. **Bourret RB**. 2010. Receiver domain structure and function in response regulator proteins. Curr Opin Microbiol **13**:142–149.
19. **Floss HG**, **Yu T-W**. 2005. Rifamycin—mode of action, resistance, and biosynthesis. Chem Rev **105**:621–632.
20. **Altschul SF**, **Madden TL**, **Schäffer AA**, **Zhang J**, **Zhang Z**, **Miller W**, **Lipman DJ**. 1997. Gapped BLAST and PSI-

BLAST: a new generation of protein database search programs. Nucleic Acids Res **25**:3389–3402.
21. **Rausch H**, **Lehmann M**. 1991. Structural analysis of the actinophage phi C31 attachment site. Nucleic Acids Res **19**:5187–5189.
22. **Gregory MA**, **Till R**, **Smith MCM**. 2003. Integration site for *Streptomyces* phage phiBT1 and development of site-specific integrating vectors. J Bacteriol **185**:5320–5323.
